# Caspase cleavage and nuclear retention of the energy sensor AMPK-α1 during apoptosis

**DOI:** 10.1016/j.celrep.2022.110761

**Published:** 2022-05-06

**Authors:** Anees Rahman Cheratta, Faisal Thayyullathil, Simon A. Hawley, Fiona A. Ross, Abdelmajdid Atrih, Douglas J. Lamont, Siraj Pallichankandy, Karthikeyan Subburayan, Ameer Alakkal, Rachid Rezgui, Alex Gray, D. Grahame Hardie, Sehamuddin Galadari

**Affiliations:** 1 Cell Death Signaling Laboratory (Division of Science), Experimental Research Building, New York University Abu Dhabi, PO Box 129188, Saadiyat Island, Abu Dhabi, UAE; 2 Core Technology Platform, Experimental Research Building, New York University Abu Dhabi, PO Box 129188, Saadiyat Island, Abu Dhabi, UAE; 3 Division of Cell Signalling & Immunology, School of Life Sciences, University of Dundee, Dundee, Scotland DD1 5EH, UK; 4 Fingerprints Proteomics Facility, School of Life Sciences, University of Dundee, Dundee, Scotland DD1 5EH, UK

**Keywords:** AMPK, cleavage, caspase, apoptosis, cl-AMPK-α1, nuclear export sequence, kinase, catalytic, anti-Fas, etoposide

## Abstract

AMP-activated protein kinase (AMPK) coordinates energy homeostasis during metabolic and energy stress. We report that the catalytic subunit isoform AMPK-α1 (but not α2) is cleaved by caspase-3 at an early stage during induction of apoptosis. AMPK-α1 cleavage occurs following Asp529, generating an ∼58-kDa N-terminal fragment (cl-AMPK-α1) and leading to the precise excision of the nuclear export sequence (NES) from the C-terminal end. This cleavage does not affect (1) the stability of pre-formed heterotrimeric complexes, (2) the ability of cl-AMPK-α1 to become phosphorylated and activated by the upstream kinases LKB1 or CaMKK2, or (3) allosteric activation by AMP or A-769662. Importantly, cl-AMPK-α1 is only detectable in the nucleus, consistent with removal of the NES, and ectopic expression of cleavage-resistant D529A-mutant AMPK-α1 promotes cell death induced by cytotoxic agents. Thus, we have elucidated a non-canonical mechanism of AMPK activation within the nucleus, which protects cells against death induced by DNA damage.

## Highlights

• AMPK-α1, but not α2, is cleaved by caspase-3 during the initial stages of apoptosis
• Cleavage of AMPK-α1 by caspase-3 occurs at Asp529 (TSLD↓S)
• Cleavage precisely removes the nuclear export sequence from the C terminus
• Cleaved AMPK-α1 accumulates in the nucleus and protects against cell death

## Graphical abstract

**Figure:**
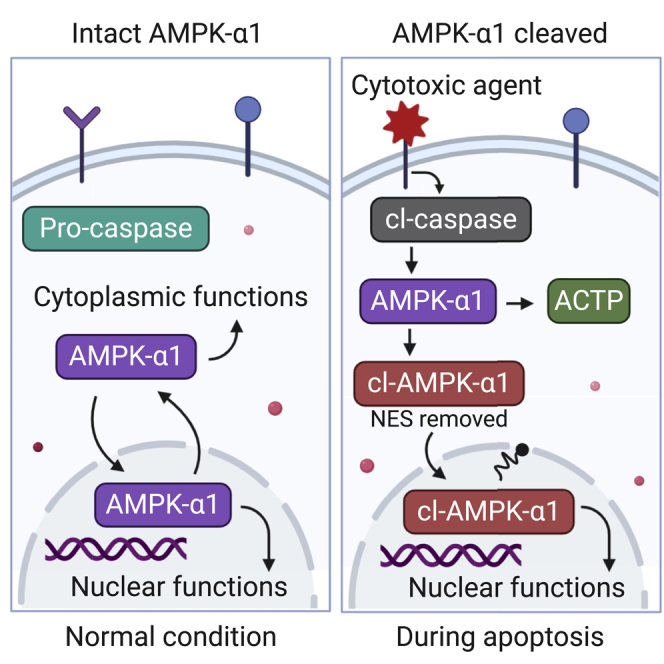

## Teaser

Cheratta et al. show that AMPK-α1 (but not α2) is cleaved by caspase-3 at Asp529 (TSLD↓S) during the initiation of apoptosis, precisely removing the nuclear export sequence from the C-terminal end. The resulting cleaved AMPK-α1 accumulates in the nucleus and protects cells against cytotoxicity induced by anticancer agents.

## Introduction

Eukaryotes have evolved sophisticated systems to maintain energy balance. A key player is AMP-activated protein kinase (AMPK), a highly conserved serine and threonine protein kinase activated by energy imbalance at both the cellular and whole-body levels (Gonzalez et al., 2020; Herzig and Shaw, 2018; Vara-Ciruelos et al., 2019, 2020). Once activated, AMPK acts to restore energy balance by switching on catabolic pathways generating ATP while switching off anabolic pathways consuming ATP. AMPK exists as heterotrimeric complexes comprising catalytic α subunits and regulatory β and γ subunits. Mammals express two α (α1 and α2), two β (β1 and β2), and three γ (γ1, γ2, and γ3) isoforms encoded by distinct genes, thus forming up to 12 heterotrimeric combinations (Ross et al., 2016). AMPK is activated by phosphorylation of a conserved threonine residue (referred to as Thr172) within the α subunit kinase domain by two distinct upstream kinases: liver kinase B1 (LKB1) and calmodulin-dependent kinase kinase-2 (CaMKK2) (Hardie, 2014; Lin and Hardie, 2017). AMPK can also be activated by displacement of ATP bound to the γ subunit by AMP or ADP. Binding of AMP, and to a lesser extent ADP, promotes AMPK activation through three distinct mechanisms: (1) promoting Thr172 phosphorylation, (2) protecting against Thr172 dephosphorylation, and (3) allosteric activation. AMPK is also activated by certain drugs, by glucose starvation, and by DNA damage via “non-canonical,” i.e., AMP- and ADP-independent, mechanisms (Lin and Hardie, 2017; Vara-Ciruelos et al., 2019, 2020).

Post-translational modifications (PTMs) greatly increase the diversity of protein function and represent key mechanisms regulating the localization, function, or stability of proteins. Changes introduced by PTMs can also affect interacting partner proteins and their downstream signal transduction pathways, thus controlling nearly every aspect of cellular regulation (Millar et al., 2019; Walsh et al., 2005). Proteolytic cleavage, whereby a peptide bond in a protein is enzymatically hydrolyzed, is an irreversible PTM often triggered by the caspase family of cysteine proteases, whose functions are inextricably linked to apoptotic cell death pathway (Julien and Wells, 2017). The number of human proteins identified as cleaved by caspases is gradually increasing, and cleavage can lead to their inhibition, activation, or functional modification. Apart from regulating morphological and biochemical features associated with apoptosis, caspase cleavage can also modulate signal transduction pathways (Julien and Wells, 2017; Timmer and Salvesen, 2007).

In the present study, we report that AMPK-α1 (but not the α2 catalytic isoform) is a target for caspase-mediated cleavage during apoptosis. Processing of AMPK-α1 by caspase-3 occurs at Asp529 (TSLD↓S), leading to the precise excision of the nuclear export sequence (NES) from the C-terminal end. The resulting N-terminal fragment (cleaved AMPK-α1 [cl-AMPK-α1]) was found to accumulate in the nucleus and to protect cells from cytotoxicity induced by anticancer agents.

## Results and discussion

### AMPK-α is cleaved during apoptosis

**Figure 1:**
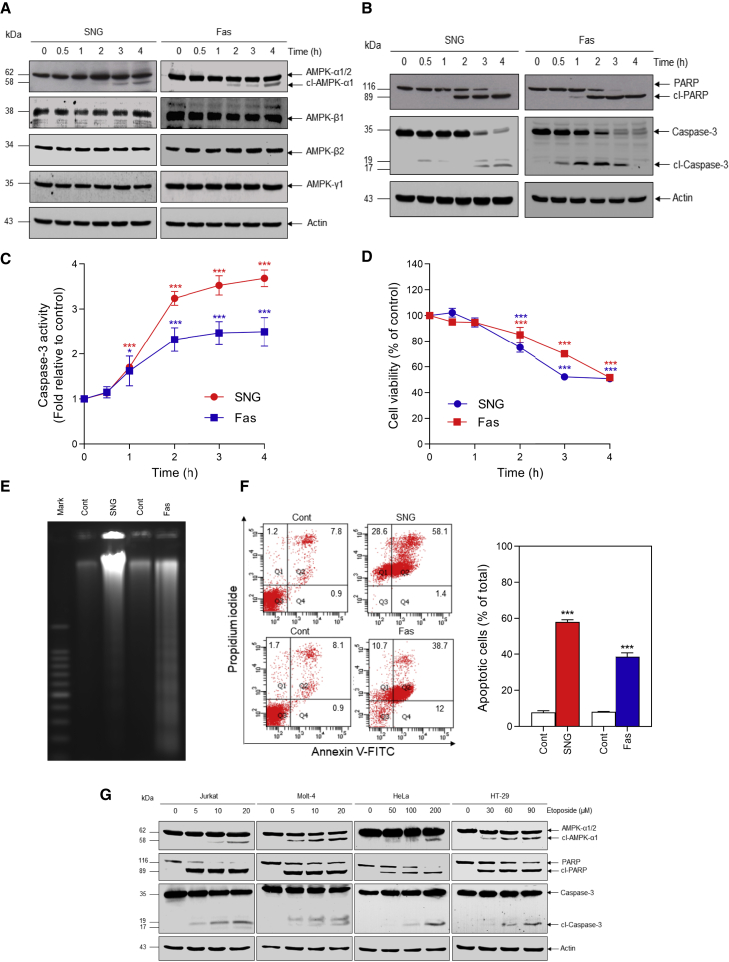
AMPK-α is cleaved during apoptosis (A and B) Jurkat cells were treated with SNG (3 μM) or anti-Fas (25 ng) for the indicated times and western blot analysis carried out. (C) Caspase-3 activity assays were performed; data shown are mean ± SD (n = 3; ^∗^p < 0.05 and ^∗∗∗^p < 0.001 versus respective control). (D) Cell viability was assessed; data shown are mean ± SD (n = 3; ^∗∗∗^p < 0.001 versus respective control). (E) Jurkat cells were treated with SNG (3 μM) or anti-Fas (25 ng) for 4 h, and DNA fragmentation assay was performed. (F) Apoptosis was assessed by annexin V-FITC/PI staining; data in the right-hand panel show mean ± SD (n = 3) for the percentage of cells positive for both annexin V binding and PI staining (^∗∗∗^p < 0.001). (G) Jurkat (6 h), Molt-4 (8 h), HeLa (24 h), or HT-29 (24 h) cells were treated with the indicated concentrations of etoposide, and western blot analysis was carried out.

We have demonstrated that sanguinarine (SNG), a benzophenanthridine alkaloid, induces apoptosis in Jurkat cells (Rahman et al., 2016). When lysates of SNG-treated Jurkat cells were probed with pan-AMPK-α1/α2 antibody, in addition to the expected 62-kDa band corresponding to the intact AMPK-α subunit, we detected a new band migrating at ∼58 kDa (Figure 1A). The new band was detectable at 2 h but was more evident after 3 to 4 h of treatment (although the 62-kDa band remained the most abundant species). An ∼58-kDa band was also detected in Jurkat cells treated with anti-Fas antibody, another apoptosis-inducing treatment (Figure 1A). Neither SNG nor anti-Fas caused significant changes in levels of the regulatory β and γ subunits (Figure 1A). Cleavage of AMPK-α induced by SNG or anti-Fas followed similar kinetics to PARP cleavage (Figure 1B), caspase activation (Figures 1B and 1C), and loss of cell viability (Figure 1D). Induction of apoptosis by SNG or anti-Fas was further confirmed using analysis of DNA fragmentation (Figure 1E) and annexin V-fluorescein isothiocyanate (FITC)/phosphatidylinositol (PI) staining (Figure 1F). To investigate whether AMPK-α cleavage is a more general phenomenon associated with apoptosis, different human cancer cell lines were treated with etoposide. Cleavage of AMPK-α and apoptosis was clearly seen following etoposide treatment in Jurkat and Molt-4 (T cell leukemia), HeLa (cervical cancer), and HT-29 (colorectal cancer) cells (Figure 1G).

### AMPK-α1 and not AMPK-α2 is specifically cleaved by caspase-3

**Figure 2:**
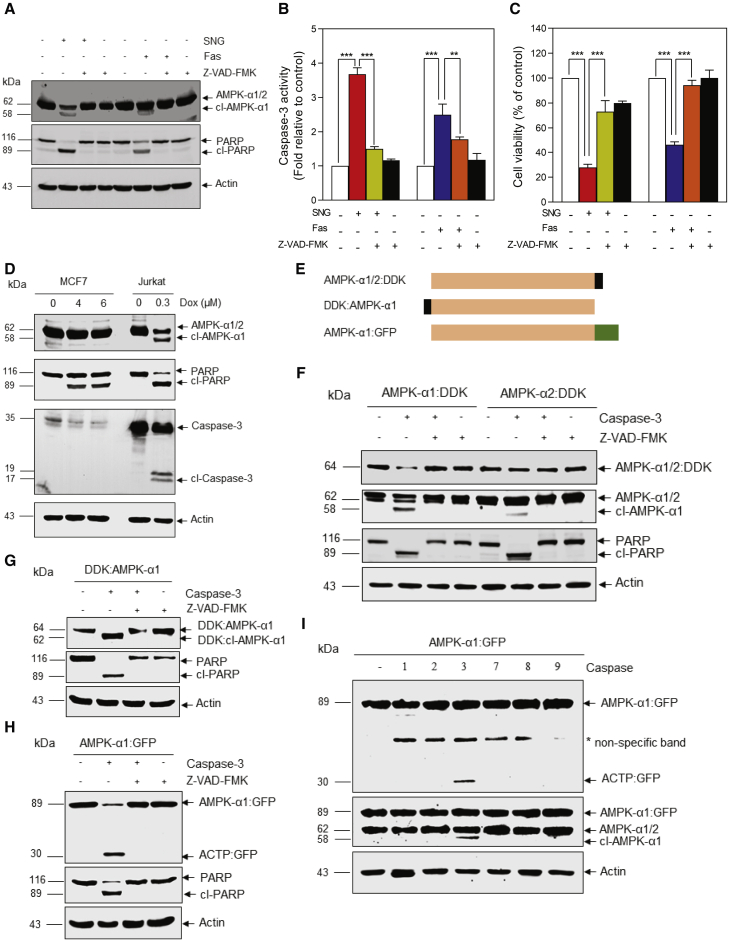
AMPK-α1 and not AMPK-α2 is specifically cleaved by caspase-3 Jurkat cells were pre-treated with Z-VAD-FMK (50 μM) for 1 h, followed by SNG (3 μM) and anti-Fas (25 ng) treatment for a further 4 h. (A–C) Western blot analysis (A), caspase-3 activity (B; data shown are mean ± SD; n = 3; ^∗∗^p < 0.01 and ^∗∗∗^p < 0.001), and cell viability (C) were carried out (data shown are mean ± SD; n = 3; ^∗∗∗^p < 0.001). (D) Western blots of lysates of MCF7 and Jurkat cells treated with indicated concentrations of Dox. (E) Schematic representation of different AMPK-α constructs. (F–H) HEK293 (F and G) and Jurkat (H) cells were transfected with the indicated plasmids, and *in vitro* caspase cleavage was performed in lysates in the presence or absence of Z-VAD-FMK (25 μM). Western blot analysis was carried out. (I) *In vitro* caspase cleavage was performed using Jurkat cell lysates containing C-terminally GFP-tagged AMPK-α1 and different caspases. Western blot analysis was carried out. Asterisk indicates non-specific band.

Caspases are among the most specific proteases involved in protein cleavage (Julien and Wells, 2017; Timmer and Salvesen, 2007). To assess their involvement in AMPK-α cleavage, we pre-incubated Jurkat cells with the pan-caspase inhibitor Z-VAD-FMK. This suppressed both SNG- and anti-Fas-induced cleavage of AMPK-α and PARP (Figure 2A), as well as caspase-3 activation (Figure 2B) and loss of cell viability (Figure 2C), indicating that caspases are responsible for AMPK-α cleavage. MCF7 breast cancer cells, which lack significant caspase-3 expression (Janicke, 2009; Wang et al., 2016), were treated with the apoptosis-inducing agent doxorubicin. Doxorubicin did not cause a detectable AMPK-α cleavage in MCF7 cells, unlike in Jurkat cells (Figure 2D), although it was able to induce apoptosis as assessed by PARP cleavage (Figure 2D).

Since AMPK-α cleavage generated an ∼58-kDa fragment from full-length 62-kDa AMPK-α, we postulated that (1) cleavage occurs close to either the N- or C-terminal end and (2) a small peptide (∼3 to 4 kDa) is separated from full-length AMPK-α by this cleavage. *In silico* analysis of the AMPK-α1 and α2 sequences did not reveal any classical DXXD caspase recognition sites, so we located the cleavage site through antibody mapping. We constructed human AMPK-α:DDK fusions in which a DDK tag was attached to the C-terminal end of AMPK-α1 and α2 (Figure 2E, upper panel). We transiently transfected these constructs into HEK293 cells and carried out *in vitro* caspase cleavage assays using active, recombinant human caspase-3. The level of AMPK-α1:DDK was significantly reduced by caspase-3, as judged by probing blots with anti-DDK antibody; cleavage of PARP and intrinsic cl-AMPK-α (detected with anti-AMPK-α1/2 antibody) were used as positive controls to confirm caspase activation (Figure 2F). Moreover, the caspase inhibitor Z-VAD-FMK prevented the reduction in AMPK-α1:DDK level and PARP cleavage, indicating that both effects were due to cleavage by caspase-3 (Figure 2F). Using anti-DDK antibody, we were unable to detect the cleaved ∼58-kDa AMPK-α1 band, suggesting that a small fragment of AMPK-α1 is cleaved from the C-terminal end. To our surprise, lysates expressing AMPK-α2:DDK fusions were not affected by caspase-3 treatment, indicating that caspase-3 cleavage is selective for AMPK-α1 rather than α2 (Figure 2F). To reveal the ∼58-kDa band, we carried out caspase cleavage *in vitro* using N-terminally tagged DDK:AMPK-α1 (Figure 2E, middle panel). As expected, incubation with caspase-3 caused generation of an ∼62-kDa DDK:cl-AMPK-α1 polypeptide from ∼64-kDa DDK:AMPK-α1, which was blocked by co-treatment with Z-VAD-FMK (Figure 2G). Based on the difference between the apparent molecular masses of full-length AMPK-α1 (62 kDa) and cl-AMPK-α1 (∼58 kDa), we anticipated that a 3- to 4-kDa fragment was removed at the C-terminal end, too small to observe using regular SDS-PAGE, even when fused to a DDK tag. To confirm this, we used a C-terminal fusion with the 27-kDa GFP protein instead (Figure 2E, lower panel), increasing the total molecular mass to ∼89 kDa. When incubated with caspase-3 and probed with anti-GFP antibody, an ∼30-kDa GFP-tagged AMPK-α1 C-terminal peptide (ACTP) was detected whose appearance was blocked by Z-VAD-FMK, confirming that the cleavage site of AMPK-α1 is indeed near the C terminus (Figure 2H). To address whether AMPK-α1 can be cleaved by other caspases, we incubated Jurkat cell lysates expressing C-terminally tagged AMPK-α1:GFP with different caspases *in vitro*. As shown in Figure 2I, AMPK-α1 was cleaved by caspase-3 but by none of the other caspases tested.

### Identification of caspase-3 cleavage site in AMPK-α1

**Figure 3:**
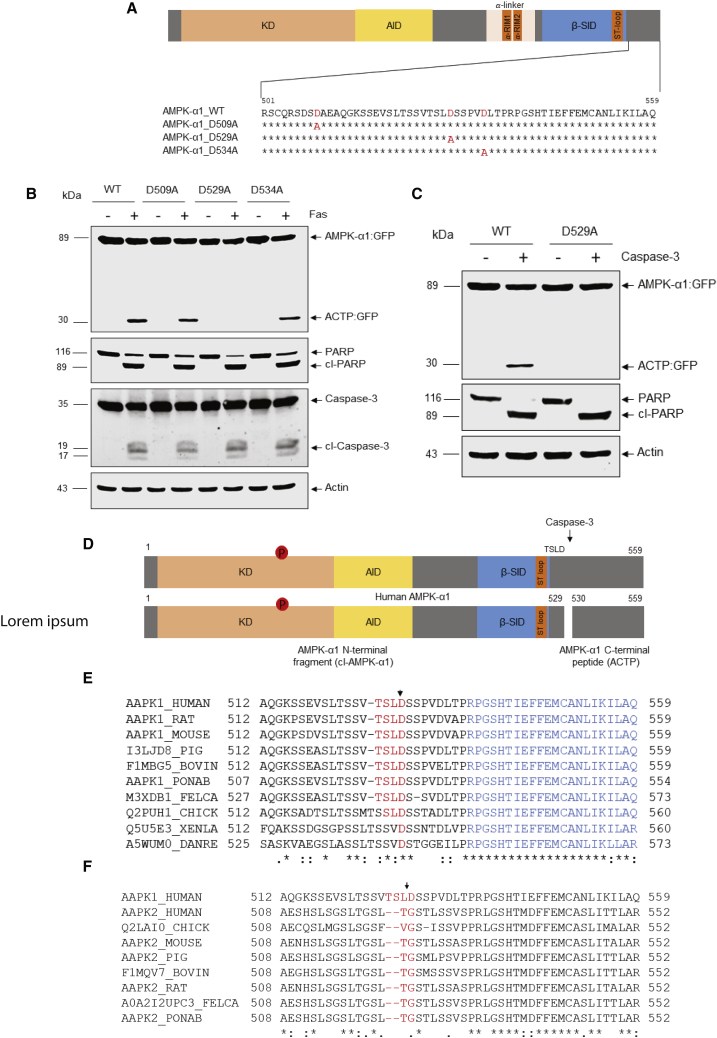
Identification of caspase cleavage site in AMPK-α1 (A) Sequences showing mutation of possible cleavage sites near C terminus of AMPK-α1. (B) Jurkat cells stably transfected with indicated constructs expressing GFP-tagged AMPK-α1 were treated with anti-Fas antibody and western blot analysis carried out. (C) *In vitro* caspase cleavage assay was performed using Jurkat cell lysates containing GFP-tagged wild type or D529A-mutant AMPK-α1. (D) Schematic diagram of AMPK-α1 cleavage by caspase. (E) Sequence alignment of AMPK-α1 in different species. Cleavage site is marked in red, and the region containing NES is marked in blue. (F) Sequence alignment of AMPK-α1 and α2 at the C-terminal tail. The cleavage site on α1 and corresponding site on α2 are marked in red and region containing NES is marked in blue.

The previous results (Figures 2F and 2H) suggested that AMPK-α1 cleavage occurs near the C-terminal end of the protein. Caspases usually cleave peptide bonds following specific aspartate residues (Julien and Wells, 2017). To map the cleavage site responsible for producing the ∼3-kDa ACTP fragment, three aspartate residues near the C-terminal end of AMPK-α1 were mutated individually to alanine (Figure 3A). Next, we stably transfected Jurkat cells with constructs expressing either GFP-tagged wild-type (WT) or mutant (D509A, D529A, and D534A) AMPK-α1. When treated with anti-Fas and probed with anti-GFP antibody, WT, D509A mutant, and D534A mutant AMPK-α1 were all cleaved to generate the ∼30-kDa ACTP:GFP fragment. However, the D529A mutant was resistant to AMPK-α1 cleavage, even with sufficient caspase activation to almost fully cleave PARP (Figure 3B). This was confirmed by *in vitro* caspase-3 cleavage assays: the D529A mutant was completely resistant to proteolytic cleavage, while the WT-AMPK-α1 was efficiently cleaved by recombinant caspase-3 (Figure 3C). Thus, AMPK-α1 is specifically cleaved by caspase-3 at the non-canonical site Asp529 (TSLD↓S) during apoptosis, generating an ∼58-kDa cl-AMPK-α1 and an ∼3-kDa ACTP (Figure 3D).

To confirm the peptide bond following Asp529 as the cleavage site by mass spectrometry (MS), we carried out cell-free assays with bacterially expressed human α1β2γ1 complex of AMPK, incubated with or without bacterially expressed, active human caspase-3 (Kang et al., 2008). Under the conditions used, the cleavage generated almost 100% conversion of full-length AMPK-α1 to cl-AMPK-α1 (Figure S1A; samples from lanes 1 and 4 were used for MS analysis). We first attempted whole-protein MS, but when digestion was performed in buffers compatible with this (low salt and no detergent or glycerol), the proteins invariably precipitated. However, we removed the precipitated proteins by centrifugation and subjected the supernatants to liquid chromatography (LC):mass spectrometry (MS). In the caspase-3-treated sample only, we detected an isotopomer family (due to the natural abundance of ^13^C; Figure S1B), corresponding to ACTP, the C-terminal peptide following Asp529 (predicted monoisotopic m/z for [M+3H]^3+^ ion, 1,105.5686). We also detected an isotopomer family with a monoisotopic mass at m/z 1,119.9044, corresponding to the same peptide but with one acetyl group added (predicted monoisotopic m/z for [M+3H]^3+^ ion, 1,119.9001). Moreover, we detected families of isotopomers (monoisotopic m/z of 829.4288 and 840.1799) corresponding to the same peptides as [M+4H]^4+^ ions (predicted monoisotopic m/z 829.4283 and 840.1769; Figure S1C). None of these were detected in the control samples incubated without caspase-3, supporting Asp529 as the cleavage site. The sequence of this peptide was fully confirmed by *de novo* sequencing using Skyline software to match the fragmentation ions (results not shown).

As further confirmation, we repeated the cleavage of the human α1β2γ1 complex with or without caspase-3 under conditions where the cleavage went almost to completion (Figure S1A, lanes 1 and 4). We separated the full-length and cleaved bands by SDS-PAGE, digested with trypsin, and analyzed the resultant peptides by LC:MS. In the control incubated without caspase-3, we obtained peptides covering 82% of the AMPK-α1 sequence, including the peptide SSEVSLTSSVTSLDSSPVDLTPR that contains Asp529 (residues 516–538) and the next tryptic peptide PGSHTIEFFEMCANLIK (539–555), located just prior to the C terminus (Table S1). The sequences of both peptides were confirmed by MS/MS fragmentation (not shown). By contrast, from cl-AMPK-α1, we obtained peptides covering 75% of the sequence, including just one matched mass for the 516–538 peptide and none for the 539–555 peptide (this compares with totals of 39 and 13 matches, respectively, with full-length AMPK-α1). The peak intensity of the 516–538 peptide isolated from full-length AMPK-α1 was also >180-fold higher than with cl-AMPK-α1, suggesting that the recovery of the peptide in the latter was due to a minor degree of cross-contamination, either during SDS-PAGE or liquid chromatography. Trypsin treatment of cl-AMPK-α1 would also be expected to produce the peptide SSEVSLTSSVTSLD (residues 516–529) terminating at Asp529. We did indeed find this peptide in both full-length and cl-AMPK-α1 (Figure S1D) samples, although the peak intensity was >150-fold higher in the cl-AMPK-α1 samples. The exact sequence of the 516–529 peptide in this sample was also fully confirmed by MS/MS fragmentation. Since trypsin would not be expected to cleave after aspartic acid, identification of this peptide further confirms the peptide bond following D529 as the cleavage site. Taken together, these MS data confirm (TSLD↓S) as the caspase-3 cleavage site, by two independent MS approaches.

The cleaved product, cl-AMPK-α1, would retain key functional domains, such as the kinase domain (KD) with the critical Thr172 phosphorylation site, the autoinhibitory domain (AID), and the flexible polypeptide α-linker (Figure 3D; Hardie, 2014; Lin and Hardie, 2017). Notably, the AMPK-α1 TSLD↓S cleavage site is conserved among vertebrates (Figure 3E) but is not present in AMPK-α2 from any vertebrate species examined (Figure 3F), explaining why cleavage is specific for the AMPK-α1 isoform.

### Biological significance of AMPK-α1 cleavage

In many cases, caspase cleavage is not just concerned with degradation of the target protein but is a purposeful, orchestrated PTM that regulates biological function. The C-terminal domain of AMPK-α interacts with that of AMPK-β, which in turn interacts with the γ subunit, thus forming the heterotrimeric complex (Hardie, 2014; Lin and Hardie, 2017). Since caspase cleaves AMPK-α1 within the C-terminal domain, we asked whether this affected its ability to form a complex with the β and γ subunits. A tricistronic plasmid encoding WT or mutant human AMPK-α1 (D529A or S530stop) plus β2 and γ1 was expressed in bacteria and any complexes formed purified using the (His)_6_ tag at the N terminus of the α1 subunit. We raised an antibody against the peptide SSPVDLTPRPGSH (referred to as “anti-ACTP” antibody), corresponding to residues 530–542 of human AMPK-α1, the first part of ACTP that is cleaved off by caspase-3. When we replaced S530 with a single TAG stop codon (S530stop), we found that a full-length α1 subunit was obtained that comigrated with the D529A mutant and was still recognized by anti-ACTP (Figure S2A). We reasoned this must have been due to readthrough of the stop codon, apparently quite common in *E. coli* (Zhang et al., 2020). In an initial attempt to overcome this, we replaced the codons for S530 and S531 with two different stop codons (TAA TAG). This produced a protein that migrated on SDS-PAGE noticeably faster than the WT and was not recognized by anti-ACTP (Figure S2B). However, there was a large amount of degradation of the α1 subunit not observed with the WT protein (Figure S2C), with fragments ranging from 30 to 45 kDa. This preparation was therefore not suitable for further analysis.

We also expressed the S530stop mutant (with a single TGA stop codon) by transient transfection in HEK293) cells carrying a double knockout of AMPK-α1 and α2. In this case, the S530stop mutant clearly migrated faster than the WT or D529A mutant. All cells expressed the AMPK-β1, β2, and γ1 subunits to similar extents (Figure S3A). However, when α1 was immunoprecipitated, the β1, β2, and γ1 subunits co-precipitated with the WT and D529A mutant, but not with the S530stop mutant, indicating that the latter did not form stable complexes with β and γ (Figure S3B). When we assayed kinase activity in anti-α1 immunoprecipitates, we detected AMP-stimulated kinase activity in immunoprecipitates from cells expressing WT or D529A, but not the S530stop mutant (Figure S3C).

**Figure 4:**
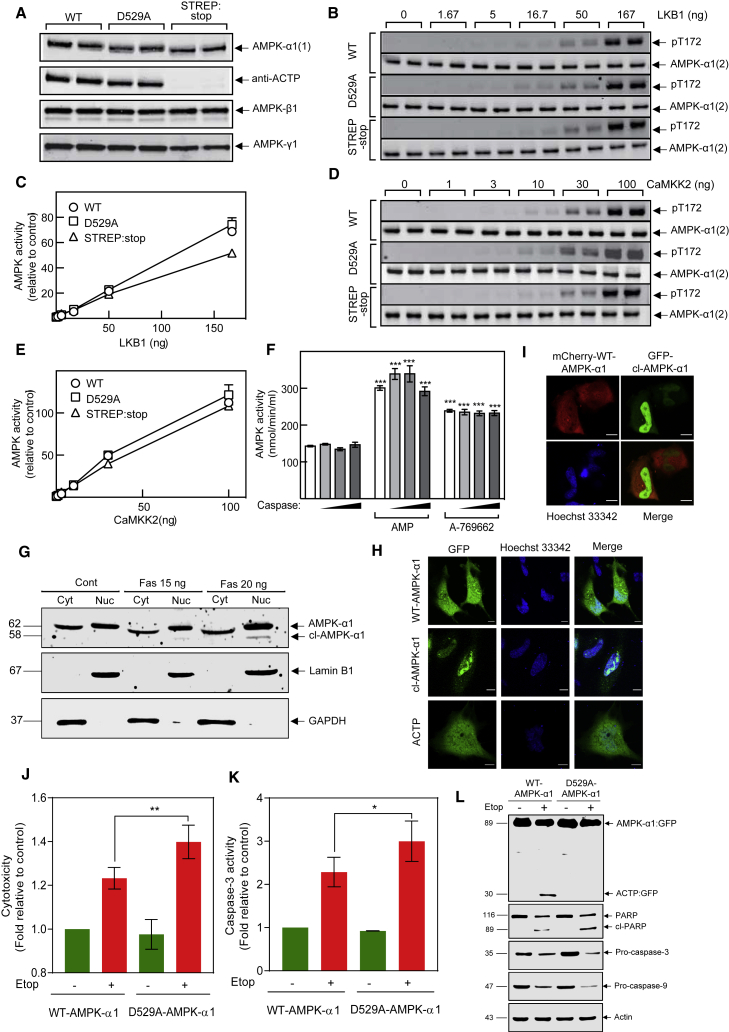
Biological significance of AMPK-α1 cleavage (A) Human α1β2γ1 complexes (WT or D529A or STREP-stop mutants) were expressed in *E. coli*; following purification on Ni^2+^-agarose (WT or D529A mutant) or Ni^2+^-agarose and Strep-Tactin columns (STREP-stop mutant), duplicate samples were analyzed by western blots probed with the indicated antibodies. (B–E) Bacterially expressed, unphosphorylated human α1β2γ1 complexes (WT, D529A, or STREP-stop) were incubated in duplicate with the indicated amounts of either (B and C) human LKB1:STRAD:MO25 complex or (D and E) CaMKK2. Western blots and AMPK activity were measured. (F) Purified rat liver AMPK was first depleted of α2-containing complexes by immunoprecipitation, and the remaining α1 complexes were incubated for 2 h with the amounts of bacterially expressed caspase-3 shown in Figure S4C. Samples were assayed for AMPK activity in the presence or absence of AMP (200 μM) or A-769662 (10 μM). Results are mean ± SEM (n = 3); significant differences from controls without AMP or A-769662 are indicated (^∗∗∗^p < 0.001). (G) Jurkat cells were treated with indicated concentrations of anti-Fas. Nuclear and cytoplasmic extractions were prepared and western blot analysis carried out. (H) HeLa cells were transiently transfected with the indicated GFP-tagged full-length (GFP-WT-AMPK-α1) or truncated (GFP-cl-AMPK-α1 and GFP-ACTP) proteins. Following transfection, cells were visualized by confocal microscopy with nuclei stained using Hoechst 33342 (scale bars, 10 μm). (I) mCherry-tagged full-length (mCherry-WT-AMPK-α1, red) and truncated (GFP-cl-AMPK-α1, green) AMPK-α1 were co-transfected into HeLa cells and cells visualized by confocal microscopy with nuclei stained with Hoechst 33342 (scale bars, 10 μm). (J–L) Jurkat cells expressing indicated constructs were exposed to etoposide (10 μM for 6 h). Cytotoxicity, caspase activity, and western blots were carried out; data shown are mean ± SD (n = 3; ^∗∗^p < 0.01 and ^∗^p < 0.05).

As a fourth approach, we returned to bacterial expression but replaced the residues after D529 with a STREP tag (the eight amino acids WSHPQFEK) followed by two stop codons (TAA TAG). This construct (termed the STREP-stop mutant) produces a polypeptide where residues 530–559 of AMPK-α1 were replaced by the STREP tag, so that undegraded full-length subunits could be purified on successive Ni^2+^-agarose and Strep-Tactin columns. This approach resulted in a complex that was recovered in rather low yield (∼5%) compared with the WT or D529A constructs. Nevertheless, when loading was normalized for equivalent amounts of α1 polypeptide, the STREP-stop α1 subunit was not recognized by anti-ACTP but purified as a complex with similar amounts of β1 and γ1 subunits as the WT and D529A mutant (Figure 4A). This STREP-stop complex, which represents a model for the cl-AMPK-α1 protein, was phosphorylated and activated in a similar manner to the WT and D529A mutants by both LKB1 (Figures 4B and 4C) and CaMKK2 (Figures 4D and 4E). Thus, the presence of the sequence from residues 530–559 of AMPK-α1 is preferred, although not essential, for the formation of active heterotrimeric complexes in bacteria but is not required for activation by phosphorylation of Thr172 by upstream kinase.

Taken together, these results suggest that α1 subunits truncated at the caspase-3 cleavage site are much less efficient at forming active heterotrimeric complexes with β and γ subunits, especially in mammalian cells. However, this did not prove that a pre-formed heterotrimeric complex would dissociate and become inactivated following cleavage by caspase-3. To address this, we studied the cleavage of AMPK complexes by caspase-3 in cell-free assays. Figure S4A shows that AMPK-α1 in highly purified rat liver AMPK (Hawley et al., 1996), or in the bacterially expressed human α1β2γ1 complex, was cleaved to similar extents by similar amounts of caspase-3. These cleavages did not affect the activity of either preparation (Figure S4B); note that the activity of recombinant human AMPK (plotted on the right Y axis) was about 30-fold lower than native rat liver kinase because it is not phosphorylated on Thr172 during bacterial expression. To address whether caspase cleavage affected allosteric activation, we focused on the rat liver preparation, because bacterial preparations always display a smaller effect. Because the rat liver preparation contains some AMPK-α2, we first removed α2 complexes by immunoprecipitation with anti-α2 antibodies and then measured the activity of the remaining AMPK-α1 complexes after treatment with various amounts of caspase-3 in cell-free assays. Figure 4F shows that allosteric activation by AMP or A-769662 were unaffected by caspase-3 treatment, despite a substantial cleavage of the α1 subunit; the β and γ subunits were unaffected by caspase cleavage (Figure S4C).

AMPK shuttles between the nucleus and cytoplasm in response to various inputs (Kodiha et al., 2007; Witczak et al., 2008), although the mechanism(s) that regulate the intracellular localization of AMPK remain poorly understood. Putative nuclear localization sequences (NLSs) have been proposed in the kinase domains of α2 and possibly α1 (Suzuki et al., 2007), although their functions as NLSs have not been confirmed (Kazgan et al., 2010). However, both AMPK-α1 and α2 have highly conserved NES in their C-terminal tails (final 22 amino acids); C-terminally truncated AMPK-α subunits lacking the NES localize to the nucleus in HEK293 cells and *Drosophila melanogaster in vivo* (Kazgan et al., 2010). The caspase-3 cleavage that we have identified is Asp529 (TSLD↓S), rather precisely removing the NES (residues 538–559; Figure 3E, marked in blue). We therefore asked whether caspase cleavage functions as a regulatory mechanism to remove the NES and restrict AMPK-α1 to the nucleus. To test this, nuclear and cytoplasmic fractions were made from anti-Fas-treated Jurkat cells and analyzed by western blotting using antibody raised against the N-terminal fragment of AMPK-α1 and α2. The majority of full-length AMPK-α was present in the cytoplasmic fraction along with the cytoplasmic marker glyceraldehyde 3-phosphate dehydrogenase (GAPDH), but cl-AMPK-α1 was observed exclusively in the nuclear fraction with the nuclear marker lamin B1 (Figure 4G). Pre-treatment with Z-VAD-FMK attenuated nuclear accumulation of cl-AMPK-α1, indicating that caspase cleavage is responsible for the AMPK nuclear accumulation (Figure S5A). Next, we examined the localization of the truncated protein by visualization of fluorescently tagged fusion protein. Expression of GFP-tagged WT-AMPK-α1, cl-AMPK-α1, and ACTP in HeLa cells and subsequent detection by confocal microscopy revealed that WT-AMPK-α1 and ACTP localized in both the cytoplasm and nucleus, whereas cl-AMPK-α1 localized predominantly in the nucleus (Figures 4H and S5B). Next, we co-transfected DNAs encoding mCherry-tagged WT-AMPK-α1 and GFP-tagged cl-AMPK-α1 in HeLa cells and confirmed a clear difference in subcellular localization (Figure 4I). These results indicate that caspase-mediated cleavage of AMPK-α1 is an intrinsic mechanism that removes the NES from AMPK-α1, inhibiting its nuclear export and causing nuclear retention.

Nuclear localization of AMPK could have many important functional consequences, including increased access to nuclear substrates, such as transcription factors and coactivators like FOXO3, PGC-1α, p53, and p300 (Greer et al., 2007; Jager et al., 2007; Okoshi et al., 2008; Yang et al., 2001), while access to cytoplasmic targets, such as acetyl-coenzyme A (CoA) carboxylase (ACC), would decrease. Furthermore, compartment-specific localization of AMPK could control its access to upstream kinases. For instance, when complexed with STRAD and MO25 (required for its kinase activity), LKB1 is excluded from the nucleus (Boudeau et al., 2003) and would thus predominantly activate AMPK in the cytoplasm. By contrast, CaMKK2 activates AMPK in both the cytoplasm and nucleus (Tsou et al., 2011; Vara-Ciruelos et al., 2018). In cell-free assays, we found that cl-AMPK-α1 and WT-AMPK-α1 were phosphorylated and activated equally well by LKB1 and CaMKK2, but this does not consider the spatial or temporal separation of AMPK and its upstream kinases within different subcellular compartments.

Previously, we demonstrated that etoposide treatment induces an LKB1-independent but CaMKK2-dependent activation of AMPK in HeLa and other cells (Vara-Ciruelos et al., 2018). Intriguingly, this only affected the α1 and not the α2 isoform and occurred exclusively in the nucleus, with no phosphorylation of the cytoplasmic AMPK target, ACC. In the present study, we show that etoposide can induce AMPK-α1 cleavage during apoptosis in HeLa cells. This cleavage may have been missed in our previous study (Vara-Ciruelos et al., 2018), because it occurs close to the C terminus and only a minor proportion of AMPK-α1 is cleaved (Figure 1G). We propose that activation of AMPK-α1 in the nucleus by etoposide is caused by cleavage by caspase-3 with consequent nuclear retention, where AMPK-α1 is phosphorylated and activated by CaMKK2 following increases in nuclear Ca^2+^. One question raised by Vara-Ciruelos et al. (2018) was why AMPK activation by etoposide was restricted to α1-containing complexes, which can now be explained because caspase cleavage is specific for the α1 isoform, with the α2 isoform lacking the TSLD↓S cleavage site (Figure 3F).

To assess the functional consequences of AMPK-α1 cleavage, Jurkat cells expressing WT-AMPK-α1 or cleavage-resistant D529A-AMPK-α1 were exposed to etoposide and cytotoxicity assessed. Interestingly, cells expressing D529A rather than WT AMPK-α1 yielded a small but significant increase in cell death from etoposide treatment, as assessed by lactate dehydrogenase (LDH) release assay (Figure 4J) and caspase activation (Figures 4K and 4L). Cells expressing D529A-AMPK-α1 treated with etoposide also demonstrated an increase in histone H2AX phosphorylation (Ser139; Figure S6A) and comet formation (Figures S6B and S6C), two important markers of DNA damage. In line with this, Vara-Ciruelos et al. (2018) demonstrated that prior CaMKK2-dependent activation of AMPK-α1 enhanced cell survival following etoposide treatment. Therefore, an AMPK inhibitor, particularly if selective for the α1 isoform, might be a useful adjunct with etoposide or other cytotoxic anticancer therapies.

What is the mechanism by which cl-AMPK-α1 protects tumor cells against the cytotoxic effects of anticancer drugs? AMPK activation by CaMKK2 causes G1 cell-cycle arrest (Fogarty et al., 2016; Vara-Ciruelos et al., 2018), reducing entry of cells into S phase, where they are more vulnerable to DNA damage induced by cytotoxic agents. Moreover, Li et al. (2019) recently demonstrated that AMPK plays a critical role in maintaining integrity of chromatin forks upon DNA replication stress. They noted a dramatic increase in AMPK-α Thr172 phosphorylation, specifically in the nucleus, after replication stress induced by either hydroxyurea or aphidicolin in HeLa and U2OS cells. Their evidence suggested that, following activation by CaMKK2, AMPK directly phosphorylated the exonuclease Exo1 at Ser746, promoting 14-3-3 binding and inhibiting Exo1 recruitment to chromatin at stalled replication forks. Thereby, AMPK could prevent excessive fork resection and consequent chromosomal instability, promoting cell survival upon replication stress. We do not exclude the possibility that cl-AMPK-α1 can play additional, as yet unidentified, roles within the nucleus. Improved understanding of compartment-specific functions of cl-AMPK-α1 will aid in the development of strategies to optimize the clinical outcome of therapeutic interventions.

In summary, AMPK-α1 is processed by caspase-3 during apoptosis from its full-length, 62-kDa form into an ∼58-kDa cl-AMPK-α1 form, which has lost the NES at the C terminus. An ∼3-kDa fragment (ACTP) is also released, which may have functions yet to be established. Our results suggest that cl-AMPK-α1 accumulates in the nucleus, where it is activated by CaMKK2, positioning it to phosphorylate nuclear targets while reducing its availability for cytoplasmic targets. As also observed by others (Li et al., 2019; Vara-Ciruelos et al., 2018), nuclear accumulation of AMPK-α plays a protective role during genotoxic stress. Our future studies will be directed toward further understanding the biological significance of AMPK-α1 cleavage in normal and cancer cells.

### Limitation of the study

We did not address in detail the biological significance of the nuclear accumulation of cleaved AMPK-α1. AMPK has been reported to phosphorylate several nuclear proteins that are either transcription factors or co-activators or that are involved in responses to DNA damage. Future studies focusing on nuclear targets phosphorylated by AMPK in response to DNA damage may help to identify novel nuclear functions of cl-AMPK-α1.

## STAR★Methods

### Key resources table

**Table:** 

REAGENT or RESOURCE	SOURCE	IDENTIFIER
**Antibodies**		

Goat anti-rabbit	Sigma	Cat#A6154; RRID:AB_258284
Goat anti-mouse	Sigma	Cat#A0412; RRID:AB_257884
Anti-actin	Santa Cruz	Cat#SC-47778; RRID:AB_2714189
Anti-AMPK-pan-α (Anti-AMPK-α1/2)	Cell Signaling	Cat#2532; RRID:AB_330331
Anti-AMPK-α1 (1)	Abcam	Cat3759; RRID:AB_304054
Anti-AMPK-α1 (2)	Raised in-house	(Woods et al., 1996)
Anti-AMPK-β1	Santa Cruz	Cat#SC-19132; RRID:AB_633859
Anti-AMPK-β2	Santa Cruz	Cat#SC-376752; RRID:AB_2861406
Anti-AMPK-pan-β	Cell Signaling	Cat#4150; RRID:AB_10828832
Anti-AMPK-ɣ1/2/3	Santa Cruz	Cat#SC-390579; RRID:AB_2861407
Anti-AMPK-ɣ1 (1)	Cell Signaling	Cat#4187; RRID:AB_10695248
Anti-AMPK-ɣ1 (2)	Raised in-house	(Cheung et al., 2000)
Anti-GAPDH	Santa Cruz	Cat#SC-47724; RRID:AB_627678
Anti-lamin B1	Santa Cruz	Cat#SC-30264; RRID:AB_2136305
Anti-GFP	Santa Cruz	Cat#SC-9996; RRID:AB_627695
Donkey anti-goat	Santa Cruz	Cat#SC-2056; RRID:AB_631730
Anti-caspase-3	Cell Signaling	Cat#9662S; RRID:AB_331439
Anti-AMPK-α1/2	Cell Signaling	Cat#2532L; RRID:AB_330331
Anti-PARP	Cell Signaling	Cat#9542L; RRID:AB_2160739
Anti-DDK	OriGene	Cat#TA50011; RRID:AB_2622345
Anti-Fas	EMD Millipore	Cat#05-201; RRID:AB_309653
Anti-H2AX	Cell Signaling	Cat#7631; RRID:AB_10860771
Anti-phospho-H2AX (Ser139)	Cell Signaling	Cat#9718; RRID: AB_2118009

**Bacterial and virus strains**		

GCI-L3	GeneCopoeia	Cat#CC003
CGI-5α	GeneCopoeia	Cat#CC001

**Chemicals, peptides, and recombinant proteins**		

3-[4,5-dimethylthiazol-2-yl]-2,5-diphenyl tetrazolium bromide (MTT)	Sigma	M5655
Dimethyl sulfoxide (DMSO)	Sigma	D5879
Hoechst 33342	Sigma	B-2883
Polybrene	Sigma	H9268
Sanguinarine (SNG)	Santa Cruz	SC-202800
Etoposide	Sigma	E1383
Doxorubicin (Dox)	Sigma	D1515
RPMI 1640 GlutaMAX	Gibco	61870-010
DMEM	Gibco	31885-023
McCoy’s 5A GlutaMAX	Gibco	36600-021
Phosphate buffered saline (PBS)	Gibco	14190-094
Fetal bovine serum (FBS)	Gibco	10270
Trypsin-EDTA	Gibco	25300-054
Penicillin/streptomycin	Gibco	15140-122
Z-VAD-FMK	Enzo	ALX-260-020-M005
Puromycin dihydrochloride	Invivogen	ANT-PR-1
AMARA peptide	Custom synthesis (Dale et al., 1995)	NA
Protease Inhibitor Cocktail, cOmplete, EDTA-free	Roche	11873580001

**Critical commercial assays**		

Active recombinant caspase set IV	Bio Vision	K233-10-25
Mega Tans 1.0 transfection reagent	OriGene	TT200003
Lenti-Pac expression packing kit	GeneCopoeia	HPK-LvTR-20 (LT001)
NE-PER™ nuclear and cytoplasmic extraction reagents	Thermo Fisher	78835
CaspACE™ Assay System	Promega	G7220
Annexin V-FITC/propidium iodide (PI) apoptosis detection kit	BD	556547
CytoTox-ONE™ homogenous membrane integrity assay	Promega	G7891
QuikChange Site-directed mutagenesis kit	Agilent Technologies	200518
CytoTox-ONE™ homogenous membrane integrity assay	Promega	G7891
Comet Assay Kit	Abcam	Ab238544

**Experimental models: Cell lines**		

Human leukemic Jurkat	ATCC	TIB-152; RRID:CVCL_0367
Human leukemic Molt-4	ATCC	CRL-1582; RRID:CVCL_0013
Human breast cancer MCF7	ATCC	HTB-22; RRID:CVCL_0031
Human cervical carcinoma HeLa	ATCC	CCL-2; RRID:CVCL_0030
Human embryonic kidney HEK293	ATCC	CRL-1573; RRID:CVCL_0045
Human colorectal carncer HT-29	ATCC	HTB-38; RRID:CVCL_0320
293Ta lentiviral packaging cell line	GeneCopoeia	Clv-PK-01 (LT008)

**Recombinant DNA**		

pCMV6-myc-DDK-AMPK-α1 with C-terminal DDK tag	OriGene	Cat#RC218572
pCMV6-myc-DDK-AMPK-α2 with C-terminal DDK tag	OriGene	Cat#RC210226
pCMV6-AN-Myc-DDK-AMPK-α1 with N-terminal DDK tag	OriGene	Cat#RC218572
pRP[Exp]-Puro-EF1A>mCherry-AMPK-α1 with N-terminal mCherry tag	VectorBuilder	VB180924-1121rhh
pRP[Exp]-Puro-EF1A>EGFP-AMPK-α1 with N-terminal GFP tag	VectorBuilder	VB180924-1122ycm
pRP[Exp]-Puro-EF1A>EGFP control vector	VectorBuilder	VB180823-1120qay
pRP[Exp]-Puro-EF1A>EGFP-AMPK-α1 (aa1-529) with N-terminal GFP tag	VectorBuilder	VB180924-1125vax
pRP[Exp]-Puro-EF1A>AMPK-α1(aa530-559)-EGFP with C-terminal GFP tag	VectorBuilder	VB180924-1128rtd
pLV[Exp]-Puro-EF1A>AMPK-α1-EGFP with C-terminal GFP tag	VectorBuilder	VB180420-1015tnn
pLV[Exp]-Puro-EF1A>AMPK-α1(D509A)-EGFP with C-terminal GFP tag	VectorBuilder	VB180420-1012dzy
pLV[Exp]-Puro-EF1A>AMPK-α1(D529A)-EGFP with C-terminal GFP tag	VectorBuilder	VB180420-1016sdf
pLV[Exp]-Puro-EF1A>AMPK-α1(D534A)-EGFP with C-terminal GFP tag	VectorBuilder	VB180420-1018gbg
Bacterial expression plasmid for GST-CaMKK2/β	(Hawley et al., 2005)	N/A
Bacterial expression plasmid for active human caspase-3	(Kang et al., 2008)	N/A
Plasmids for expression of human (His)_6_-tagged LKB1, MO25α and STRADα in insect (Sf9) cells	(Zeqiraj et al., 2009)	N/A
Tricistronic plasmic for expression of human AMPK complex (α1β2γ1) in bacteria	University of Dundee (https://sls-reagents.co.uk/)	Cat# DU32489

**Software and algorithms**		

Prism 8.3	Graph Pad	RRID:SCR_005375
FACSDiva	BD	RRID:SCR_001456
ImageJ	NIH	RRID:SCR_003070
Fiji	Fiji	RRID:SCR_002285
ClustalW	EMBL-EBI	RRID:SCR_017277
Mascot	Matrix Science	RRID:SCR_014322

### Resource availability

#### Lead contact

Further information and requests for reagents may be directed to and will be fulfilled by the Lead Contact, Sehamuddin Galadari (sehamuddin@nyu.edu).

#### Materials availability

Plasmid DNA constructs used in this study were from Origene Technologies and VectorBuilder. Tricistronic plasmids for expression of human AMPK complex (α1β2γ1) in bacteria, and anti-ACTP antibody can be obtained directly from the University of Dundee.

### Experimental model and subject details

#### Cell lines, cell culture conditions, and drug treatment

Human leukemic Jurkat and Molt-4 cells and human breast cancer MCF7 cells were grown in RPMI 1640 GlutaMAX medium. Human cervical carcinoma HeLa cells and human embryonic kidney HEK293 cells were grown in DMEM medium. Human colorectal carcinoma HT-29 cells were grown in McCoy’s 5A GlutaMAX medium (Cells were bought from ATCC, VA, USA). Cells were grown in respective medium supplemented with 10% v/v heat-inactivated FBS, 25 IU/mL penicillin, and 25 μg/mL streptomycin in an incubator containing humidified atmosphere of 95% air and 5% CO_2_ at 37°C. SNG (10 mM), etoposide (20 mM), and Dox (10 mM) were prepared in DMSO. Anti-Fas antibody (0.5 mg/mL) was directly used for the treatment. For the treatment, cells were grown to about 80% confluence, and then exposed to the desired concentrations of drugs for the required time period. Cells grown in a medium containing an equivalent amount of DMSO without drugs served as control.

#### Plasmids and transient transfection

pCMV6-myc-DDK-AMPK-α1 with C-terminal DDK tag (RC218572), pCMV6-myc-DDK-AMPK-α2 with C-terminal DDK tag (RC210226), and pCMV6-AN-Myc-DDK-AMPK-α1 with N-terminal DDK tag (RC210226) were purchased from OriGene Technologies, Inc (Rockville, MD, USA). pRP[Exp]-Puro-EF1A>mCherry-AMPK-α1 with N-terminal mCherry tag (VB180924-1121rhh), pRP[Exp]-Puro-EF1A>EGFP-AMPK-α1 with N-terminal GFP tag (VB180924-1122ycm), pRP[Exp]-Puro-EF1A>EGFP control vector (VB180823-1120qay), pRP[Exp]-Puro-EF1A>EGFP-AMPK-α1 (aa1-529) with N-terminal GFP tag (VB180924-1125vax), and pRP[Exp]-Puro-EF1A>AMPK-α1(aa530-559)-EGFP (VB180924-1128rtd) with C-terminal GFP tag were purchased from Vector Builder Inc. (Chicago, IL, USA). DNA transfection was performed by using Mega Tans 1.0 transfection reagent from OriGene Technologies, Inc (Rockville, MD, USA) as described in the manufacture’s protocol.

#### Establishment of the stable jurkat cell line expressing AMPK-α1 and mutants

C-terminal GFP tagged-pLV[Exp]-Puro-EF1A>AMPK-α1-EGFP (VB180420-1015tnn), -pLV[Exp]-Puro-EF1A>AMPK-α1(D509A)-EGFP (VB180420-1012dzy), -pLV[Exp]-Puro-EF1A>AMPK-α1(D529A)-EGFP (VB180420-1016sdf), and -pLV[Exp]-Puro-EF1A>AMPK-α1(D534A)-EGFP (VB180420-1018gbg) were purchased from VectorBuilder Inc. (Chicago, IL, USA). In order to generate lentiviral particle, plasmids were individually co-transfected with packaging vectors in 293Ta lentiviral packaging cell line (Clv-PK-01) (GeneCopoeia, Rockville, MD, USA) using Lenti-Pac expression packing kit (HPK-LvTR-20) (GeneCopoeia, Rockville, MD, USA). Jurkat cells were infected with virus-containing medium (supplemented with 4 μg/mL Polybrene). The transduced cells were selected with puromycin dihydrochloride (0.3 μg/mL) for 7–14 days to generate stable cell lines. Positive clones were confirmed by Western blot analysis.

#### Site-directed mutagenesis

The mutant clones were generated commercially by site-directed mutagenesis of C-terminal GFP tagged-pLV[Exp]-Puro-EF1A>AMPK-α1-EGFP (VB180420-1015tnn) purchased from Vector Builder Inc. (Chicago, IL, USA). The Asp residues at positions 509 (SDSD509), 529 (TSLD529), and 534 (SPVD534) were replaced by Ala. The resulting mutants were denoted as D509A, D529A, and D534A, respectively. The mutations were confirmed by sequencing analysis.

#### Purification of heterotrimeric AMPK complex from bacteria

Polycistronic bacterial expression plasmids encoding (His)_6_-tagged human AMPK-α1, plus AMPK-β2 and -γ1 were used to inoculate LB auto-induction media at 37°C. When A_600nm_ was ≈0.5 the culture was switched to 25°C and expression continued for a further 16 h. The culture was harvested by centrifugation, lysed a using pestle and mortar, resuspended in 50 mM Tris-HCl, pH 8.0, 500 mM NaCl, 20 mM imidazole, plus protease inhibitor cocktail (Roche) and clarified by centrifugation. The supernatant was applied to a 5 mL HisTrap column (Cytiva) and eluted using gradient from 20 to 500 mM imidazole (30 mL). The eluted protein was dialysed into 50 mM Na Hepes, pH 8.0, 150 mM NaCl.

Mutations in human AMPK-α1 (D529A, S530stop, S530stop-S531stop, STREP-stop) were made in the polycistronic expression plasmid using the QuikChange Site-Directed Mutagenesis kit and expressed as above. The STREP-stop mutant was made using three rounds of mutagenesis. Following purification on the HisTrap column as above, it was further purified on a Strep-trap HP column (1 mL, Cytiva) and bound protein eluted in 50 mM Hepes, pH 7.4, 200 mM NaCl, 2.5 mM desthiobiotin.

#### Expression of the WT, D529A, and S530stop mutant in HEK293 cells

A pcDNA5 FRT vector encoding human AMPK-α1 with a C-terminal FLAG tag (Hawley et al., 2014) was used as a template to create D529A and S530stop mutants using the QuikChange Site-Directed Mutagenesis kit; mutations were confirmed by sequencing. AMPK α1^−/−^ α2^−/−^ (double knockout) HEK293 cells were generated using the CRISPR-Cas9 method as previously described for G361 cells (Fogarty et al., 2016). They were transiently transfected with DNAs encoding FLAG-tagged wild type AMPK-α1, the D529A or the S530stop mutant, using FuGENE 6 according to manufacturers’ instructions. After transfection for 48 h, cell lysates were prepared (Hawley et al., 2018).

#### Expression of other proteins

CaMKK2 was expressed as an N-terminal glutathione-S-transferase fusion (Hawley et al., 2005). Briefly, bacterial cells expressing the construct were used to inoculate LB auto-induction media at 37°C. When A_600nm_ ≈ 0.5 the culture was switched to 20°C and expression continued for a further 16 h. The culture was harvested by centrifugation, lysed using a pestle and mortar, resuspended in 50 mM Tris-HCl, pH 8.0, 500 mM NaCl, 1 mM DTT, 1 mM EDTA, 1 mM EGTA and protease inhibitor cocktail and clarified by centrifugation. The supernatant was applied to a 5 mL GST FF column (Cytiva) and eluted in 50 mM Na Hepes, pH 8.0, 200 mM NaCl and 20 mM glutathione.

His-tagged LKB1:STRAD:MO25 was expressed in insect (Sf9) cells (Zeqiraj et al., 2009). The resulting pellet was lysed using a pestle and mortar, resuspended in 50 mM Tris-HCl, pH 8.0, 500 mM NaCl, 20 mM imidazole and protease inhibitor cocktail, and clarified by centrifugation. The supernatant was applied to a 5 mL HIS Trap column (Cytiva) and eluted using a gradient from 20 to 500 mM imidazole (30 mL). The eluted protein was concentrated and applied to a Superdex 200 gel filtration column equilibrated in 50 mM Na Hepes, pH 7.5, 200 mM NaCl.

Plasmid expressing His-tagged active caspase-3 (Kang et al., 2008) was a kind gift from Sang Jeon Chung at Sungkyunkwan University. Briefly, bacterial cells expressing the construct were used to inoculate LB auto-induction media at 37°C. When A_600nm_ ≈ 0.5 the culture was switched to 16°C and expression continued for a further 24 h. The culture was harvested by centrifugation, lysed using a pestle and mortar, resuspended in 50 mM Tris-HCl, pH 8.0, 500 mM NaCl, 20 mM imidazole and protease inhibitor cocktail and clarified by centrifugation. The supernatant was applied to a 5 mL HIS Trap column (Cytiva) and eluted using a gradient from 20 to 500 mM imidazole (30 mL). The eluted protein was concentrated and dialysed into 50 mM Na Hepes, pH 7.5, 50 mM NaCl.

#### Generation of anti-α1-C-terminal peptide (anti-ACTP) antibody

The antibody against the C-terminal region of AMPK-α1 was raised in sheep using the synthetic peptide CSSPVDLTPRPGSH (residues 530–542 of human AMPK-α1, with an N-terminal cysteine to allow coupling via the thiol to keyhole limpet hemocyanin). The coupling and immunization procedures, and affinity purification of the antibody, were as described previously (Woods et al., 1996).

### Method details

#### Protein lysate preparation and Western blot analysis

After the treatment with the required concentrations of drugs for indicated time-period, cells were washed with PBS, whole-cell lysates were prepared, and Western blot analysis was performed as described previously. Cytoplasmic and nuclear fractions were extracted using NE-PER™ nuclear and cytoplasmic extraction reagents (Thermo Fisher Scientific, Waltham, MA, USA)

#### Enzymatic caspases-3 assay

The enzymatic activity of caspase-3 following SNG and anti-Fas treatment were measured according to the manufacture protocol (CaspACE™ Assay System; Promega). Briefly, after the drug treatment, cells were lysed in 200 μL of lysis buffer (50 mM HEPES, pH 7.4, 5 mM CHAPS, and 5 mM DTT) by freeze and thawing. The cell lysates were centrifuged (20,000 g for 15 min), 50 μg of protein was incubated with 30 μL of caspase assay buffer and 2 μL of caspase-3 colorimetric substrate (DEVD-pNA) at 37°C for 3 h. The optical density of the reaction mixture was quantitated at 405 nm by using EnSpire microplate reader (PerkinElmer, MA, USA).

#### MTT cell viability assay

Cells grown to about 80% confluence were treated with the required concentration of drugs. After the treatment, 25 μL of MTT (5 mg/mL) per 100 μL media was added to each well and incubated for 4 h at 37°C. The formazan crystals formed were dissolved in DMSO and absorbance was measured at 570 nm using EnSpire microplate reader (PerkinElmer, MA, USA). The cytotoxicity was expressed as percentage over control.

#### LDH release assay

Jurkat cells (3.5 × 10^6^ cells per plate) treated with etoposide (10 μM for 6 h) were subjected to LDH release assay as per manufactures protocol (CytoTox-ONE™ homogenous membrane integrity assay). Briefly, 100 μL of culture media were incubated with equal volume of CytoTox-ONE reagent for 10 min at 22°C. Reaction was stopped by adding 50 μL of stopping reagent. Fluorescence was recorded with an excitation wavelength of 560 nm and an emission wavelength of 590 nm.

#### Comet assay

Jurkat cells (3.5 × 10^6^ cells per plate) treated with etoposide (10 μM for 6 h) were subjected to comet assay as per manufactures protocol (Comet Assay Kit; Abcam). Briefly, cells were centrifuged at 700 × g for 2 min and discard supernatant. Pellet were washed and resuspend at 1 × 10^5^ cells/mL with ice-cold PBS (without Mg^2+^ and Ca^2+^. Cell samples were mixed with comet agarose at 1/10 ratio (v/v) and 75 μL is immediately transferred onto previously prepared agarose base layer slide. Slides were immersed in pre-chilled lysis buffer for 30–60 min at 4°C in the dark followed by pre-chilled alkaline solution for 30 min at 4°C in the dark. Slides were then subjected to electrophoresis using TBE electrophoresis solution for 10–15 min at 1 volt/cm. After washing with distilled water, slides were immersed in cold 70% ethanol for 5 min and allowed to air dry. Slides were then stained with vista green DNA dye for 15 min and images were captured using IX73 inverted fluorescent microscope (Olympus, Tokyo, Japan) with a FITC filter. DNA damage was quantified using comet assay IV software (Instem, UK). The results were recorded as the tail moment over 60 cells.

#### Cleavage of purified AMPK complexes by caspase 3 in cell-free assays Figure S4

For Figure S4, bacterially expressed AMPK (900 ng; α1β2γ1 complex) or an equivalent amount of AMPK purified from rat liver (Hawley et al., 1996) were incubated with the concentrations of caspase-3 indicated for 2 h at 37°C in a reaction containing 50 mM Na Hepes, pH 7.2, 50 mM NaCl, 0.1% CHAPS, 10 mM EDTA, 5% glycerol and 10 mM dithiothreitol. After 2 h, aliquots were removed for kinase assay and Western blotting.

#### Preparation of caspase-3-cleaved AMPK for mass spectrometry

Bacterially expressed AMPK (15 μg; α1β2γ1 complex) was incubated with 15 Units of bacterially expressed caspase-3 at 37°C in a 20 μL reaction volume containing 50 mM Na HEPES, pH 7.2, 50 mM NaCl, 0.1% Triton X-100. At 2 h it was clear that the bulk of the protein had aggregated and precipitated, so the suspension was centrifuged (16,000 × g; 5 min). The supernatant was incubated in 70% methanol for 20 min at −20°C and centrifuged again (16,000 × g; 10 min). This supernatant was dried down, resuspended in 20 μL of 1% formic acid and 10 μL used for LC-MS analysis.

#### Preparation of caspase-3-cleaved AMPK for tryptic peptide analysis by mass spectrometry

Bacterially expressed AMPK (1 μg; α1β2γ1 complex) was incubated with 5 units of bacterially expressed caspase-3 at 37°C in a 20 μL volume containing 50 mM Na HEPES, pH 7.2, 50 mM NaCl, 0.1% CHAPS, 10 mM EDTA, 5% glycerol and 10 mM DTT. After 2 h, the reaction was terminated by the addition of 4 × SDS sample buffer (Invitrogen) containing 1 mM DTT.

#### LC:MS analysis

This was performed by the FingerPrints Proteomics Facility (University of Dundee). Analysis of peptides used a Q Exactive plus Mass Spectrometer (Thermo Scientific) coupled with a Dionex Ultimate 3000 RS (Thermo Scientific). Buffers for LC were: buffer A (0.1% (v/v) formic acid), buffer B (80% (v/v) acetonitrile and 0.1% (v/v) formic. Sample aliquots (10 μL) were loaded at 10μL/min onto a trap column (100 μm × 2 cm, PepMap nanoViper C18 column, 5 μm, 100 Å, Thermo Scientific) which was equilibrated with 0.1% trifluoroacetic acid. The trap column was washed for 3 min at the same flow rate and then switched in-line with a Thermo Scientific resolving C18 column maintained at 50°C (75 μm × 50 cm, PepMap RSLC C18 column, 2 μm, 100 Å). Peptides were eluted at 300 nL/min with a linear gradient from 2% buffer B to 5% buffer B over 3 min, 5% buffer B to 35% buffer B over 64 min, and then to 98% buffer B over 2 min. After each run the column was washed with 98% buffer B for 20 min and re-equilibrated in 2% buffer B for 17 min.

The source voltage for MS was 2.3 Kv and the capillary temperature 250°C. A scan cycle comprised MS1 scan (m/z range from 350–1600, ion injection time of 20 ms, resolution 70,000 and automatic gain control (AGC) 1 × 10^6^) acquired in profile mode, followed by 15 sequential dependent MS2 scans (resolution 17500) of the most intense ions fulfilling predefined selection criteria (AGC 2 × 10^5^, maximum ion injection time 100 ms, isolation window of 1.4 m/z, fixed first mass of 100 m/z, spectrum data type: centroid, intensity threshold 2 × 10^4^, exclusion of unassigned, singly and >5 charged precursors, peptide match preferred, exclude isotopes on, dynamic exclusion time 45 s). The HCD collision energy was set to 27% of the normalized collision energy. Mass accuracy was confirmed before sample analysis. Two blanks were run between each sample to reduce carry-over.

#### DNA fragmentation analysis

Jurkat cells (3.5×10^6^ cells per plate) treated with SNG and anti-Fas antibody were washed with PBS, and incubated with 200 μL of lysis buffer (50 mM Tris-HCl at pH 7.5, 3% nonionic detergent IGEPAL CA-630, and 20 mM EDTA) for 10 min. The lysates were used to perform the DNA fragmentation analysis as described previously (Thayyullathil et al., 2013). Briefly, after the treatment, cells were washed with PBS and incubated with 200 μL of lysis buffer (50 mM Tris–HCl - pH 7.5, 3% non-ionic detergent IGPAL CA-630, and 20 mM EDTA) for 10 min. The samples were centrifuged at 1000xg for 5 min in order to collect the supernatant which contain apoptotic DNA fragment. SDS (10 μL, 20%) was added and the supernatants were incubated with 0.4 μg/mL RNase at 56°C for 2 h to remove the cellular RNA. Proteinase K (1.5 μg/mL) was then added to the supernatant at 56°C and it was further incubated for 2 h to remove the proteins. The DNA was then precipitated with 0.1 volume of 3 M sodium acetate and 2.5 volume of ice-cold absolute ethanol. After centrifugation, the DNA pellet was washed with 70% ethanol and then air dried. The dried pellet was re-suspended in 20 μL TE buffer (10 mM Tris–HCl - pH 7.5 and 0.1 mM EDTA) and incubated at 65°C for 5 min. Finally, the resuspended DNA was subjected to electrophoresis on a 2% agarose gel at a constant voltage of 40 V for 1–2 h.

#### Quantification of apoptosis using FACS analysis

SNG- or anti-Fas-induced apoptosis was quantified by using Annexin V-FITC/propidium iodide (PI) apoptosis detection kit (BD Pharmingen, USA) according to the manufacturer’s protocol. Briefly, after the treatment, cells were harvested, washed with PBS, and stained with Annexin V-FITC and PI. The level of apoptosis was quantified using BD FACSCanto II and FACSDiva software (BD Biosciences, NJ, USA).

#### *In vitro* caspase cleavage assay

Cell lysates (50 μg of total protein) were incubated for 2 h at 37°C in PIPES (piperazine-N,N′-bis 2-ethanesulfonic acid) assay buffer (20 mM PIPES, 100 mM NaCl, 10 mM dithiothreitol, 1 mM EDTA, 0.1% wt/vol CHAPS, and 10% wt/vol sucrose - pH 7.2) containing 10 units of recombinant active caspase(s) in the presence or absence of a caspase inhibitor (25 μM). The incubation was terminated with the addition of an equal volume of 2× sample buffer (100 mM Tris-HCl - pH 6.8, 200 mM DTT, 4% wt/vol SDS, 20% vol/vol glycerol, and 0.2% bromphenol blue). Protein cleavage was assessed by Western blotting.

#### Phosphorylation of bacterially expressed AMPK by LKB1 or CaMKK

Bacterially expressed human AMPK (α1β2γ1 complex; unphosphorylated on threonine 172; 500 ng) was incubated for 10 min in a total volume of 25 μL with 200 μM ATP and 5 mM MgCl_2_ in the presence of the indicated amount of either the human LKB1:STRAD:MO25 complex or CaMKK2. Aliquots were removed for Western Blotting and kinase assays.

#### Kinase assays of AMPK

Kinase assays for AMPK were carried out as described previously (Fyffe et al., 2018). Briefly, the transfer of radioactivity from [γ-^32^P] ATP to a synthetic peptide was determined by binding the peptide to squares of P81 phosphocellulose paper and washing with 1% (v/v) phosphoric acid. As discussed in Fyffe et al. (2018), the *AMARA* peptide was used except where allosteric activation was being measured, when the *SAMS* peptide was used.

#### Confocal microscopy

HeLa cells (0.6×10^6^ cells per plate) seeded in to glass bottom plates were transiently transfected with corresponding plasmids. 24 h after the transfection, cells were washed in PBS, incubated for 15 min with DNA specific fluorescent dye, Hoechst 33342 (1.5 mL; 10 mg/mL stock solution) at 37°C. The stained cells were washed twice with PBS and images were acquired using Olympus FV1000 upright confocal microscope with excitation at 488 nm for GFP and 405 nm for Hoechst and Emission range set from 500 to 550 nm for GFP and from 415–465 nm for Hoechst on the spectral detector. 30 cells were analyzed using ImageJ: the blue channel image was used as a mask to determine the location of the nucleus in the green channel. An ROI was then drawn in the nucleus and in the cytoplasm and average intensities were calculated and their ratio plotted.

### Quantification and statistical analysis

Statistical analysis was performed using Graph Pad Prism 8.3 software. Data are shown as mean ± standard deviation or standard error of mean (*n* = 3) except otherwise specified. Significance was analyzed by using One-way ANOVA (Bonferroni post hoctest test) or unpaired t test. Asterisk (^∗^) represents p value < 0.05, double-asterisk (^∗∗^) represents p value < 0.01, and triple-asterisk (^∗∗∗^) represents p value < 0.001. A difference was considered significant when p < 0.05.

## Data Availability

• All data reported in this paper will be shared by the lead contact upon reasonable request. • This paper does not report original code. • Any additional information required to reanalyze the data reported in this paper is available from the lead contact upon request.

## Supplemental information

Document S1. Figures S1–S6 and Table S1

Document S2. Article plus supplemental information
