# Supplementary material for: Caspase cleavage and nuclear retention of the energy sensor AMPK-α1 during apoptosis
Source: Cell Rep. 2022 May 6;39(5):110761. doi: 10.1016/j.celrep.2022.110761 (PMC9108549; doi:10.1016/j.celrep.2022.110761)
Supplement: Document S1. Figures S1–S6 and Table S1 [file mmc1.pdf]

Cell Reports, Volume *39*

Supplemental information

Caspase cleavage and nuclear retention of the energy sensor AMPK-α1 during apoptosis

Anees Rahman Cheratta, Faisal Thayyullathil, Simon A. Hawley, Fiona A. Ross, Abdelmajdid Atrih, Douglas J. Lamont, Siraj Pallichankandy, Karthikeyan Subburayan, Ameer Alakkal, Rachid Rezgui, Alex Gray, D. Grahame Hardie, and Sehamuddin Galadari

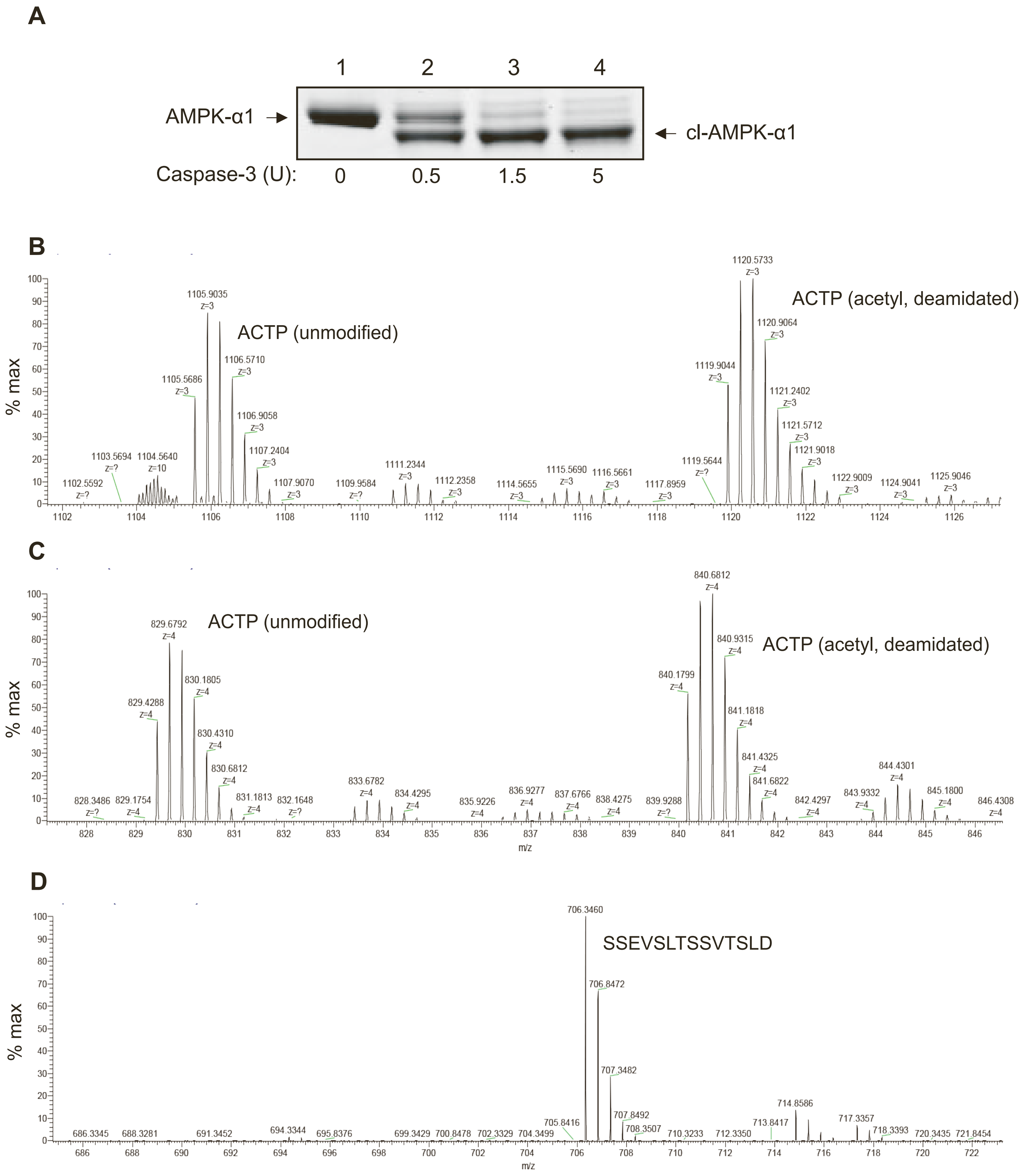

Figure S1. Identification of caspase-3 cleavage site on the α1 subunit of the human α1β2γ1 complex by mass spectrometry, related to Figure 3. (A) Cleavage of bacterially expressed α1β2γ1 AMPK complex by bacterially expressed human caspase-3 in cell-free assays. AMPK was incubated with the indicated amounts of caspase-3 for 2 h and analyzed by SDS-PAGE. (B) Recovery of ACTP peptide $(M+3H)^{3+}$ ion. Part of mass spectrum of the small peptide (ACTP) released by caspase-3 cleavage of the α1β2γ1 complex in cell-free assays, showing $(M+3H)^{3+}$ ions. (C) Recovery of ACTP peptide $(M+4H)^{4+}$ ions. Part of mass spectrum of the small peptide (ACTP) released by caspase-3 cleavage of the α1β2γ1 complex in cell-free assays, showing $(M+4H)^{4+}$ ions. (D) Recovery of peptide SSEVSLTSSVTSLD $(M+2H)^{2+}$ from caspase-treated sample. Part of mass spectrum of peptides derived by tryptic digestion of cl-AMPK-α1 (generated by caspase-3 cleavage of the human α1β2γ1 complex) showing the peptide SSEVSLTSSVTSLD.

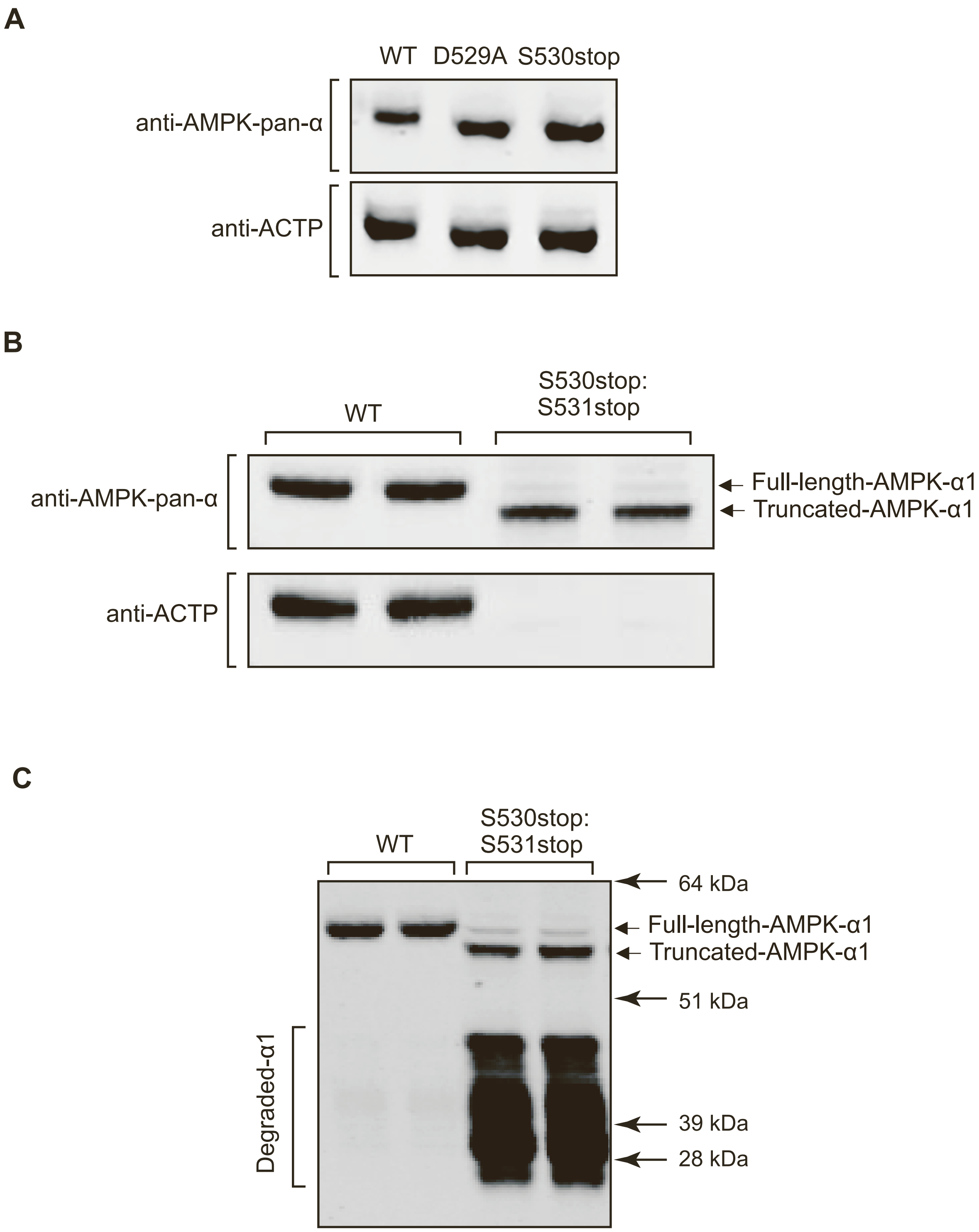

Figure S2. S530stop:S531stop mutation caused a large amount of degradation of the α1 subunit, related to Figure 4. (A) Bacterial expressed AMPK multimeric complex. A tricistronic plasmid expressing His-tagged AMPK-α1, AMPK-β2, and AMPK-γ1 was expressed in bacteria and purified using-Ni2+-agarose. The α1 subunit had either the wild type sequence (WT), or carried a D529A or S530stop mutation (the latter with a single TGA stop codon in place of S530). Purified proteins were analyzed by Western blotting and probed using anti-AMPK-pan-α or an in-house antibody raised against the cleaved ACTP sequence. (B) As Fig S2A except that the codons for S530 and S531 were replaced by two stop codons (TAA TAG) to create the S530stop:S531stop mutant (C) As Fig S2B except that the lower portion of the gel containing polypeptides with molecular masses from 20 kDa upwards was also analyzed, by blotting with anti-AMPK-pan-α, to reveal α-subunit degradation. Migration of molecular mass markers is indicated on the right-hand side. Note that in both Figs. S2B and S2C the loading of the S530stop:S531stop mutant had been increased so that the truncated (58 kDa) α1 subunit gave a similar signal to the WT control.

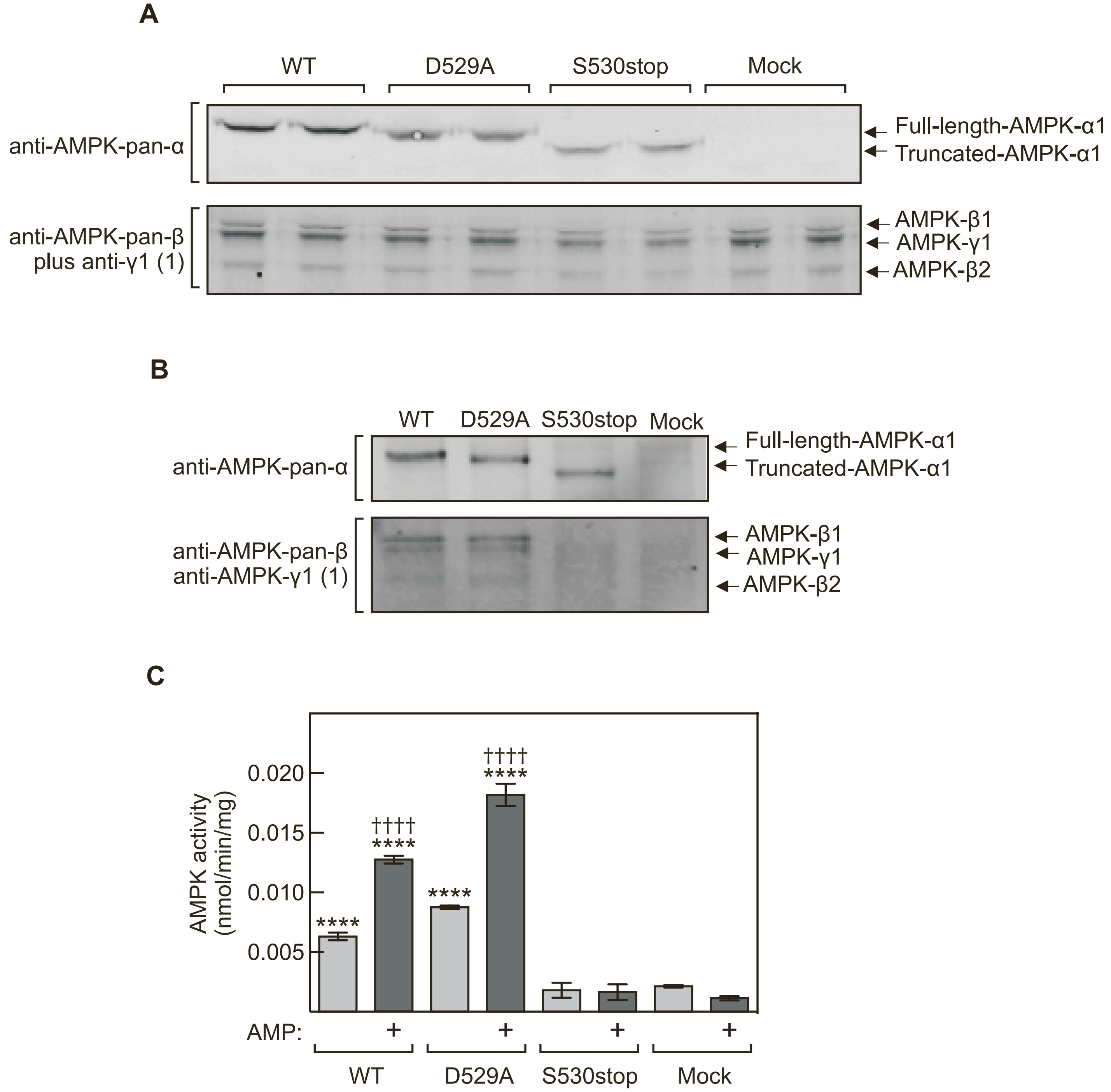

Figure S3. Transiently expressed S530stop mutant (with a single TGA stop codon) did not form stable complexes with β-γ and did not elicit AMP-stimulated kinase activity, related to Figure 4. (A) Lysates of $\alpha1^{-/-}$ $\alpha2^{-/-}$ HEK293 cells transfected with DNA encoding AMPK-pan-α (WT, D529A or S530stop), or mock-transfected control cells, were analysed by Western blotting with anti-α1 (top) or a mixture of anti-β1/β 2/γ1 antibodies (bottom) (B) As Fig S3A, except that samples of lysate were immunoprecipitated with anti-α1 antibodies prior to Western blotting. (C) As Fig S3B, except that the immunoprecipitates were analyzed by AMPK kinase assay in the presence or absence of 200 μM AMP. Results are mean ± SEM (n = 3). Significant differences from mock-transfected controls are indicated by asterisks, and from assays without AMPK by daggers.

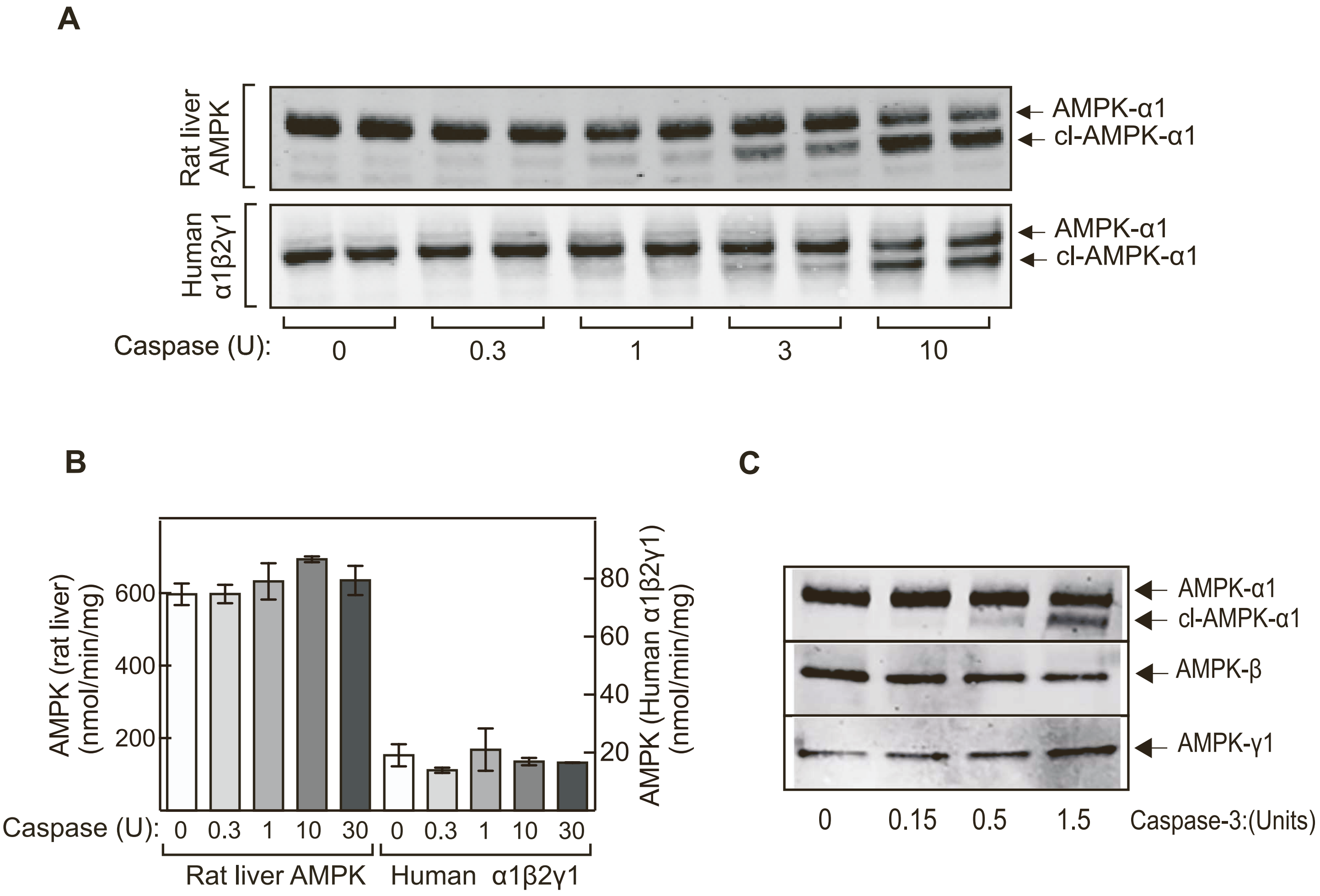

Figure S4. Cleavage of AMPK complexes by caspase-3 in cell-free assays, related to Figure 4. (A) Cell-free cleavage of purified rat liver AMPK and human α1β2γ1 complex. AMPK preparations were incubated in duplicate for 1 h with the indicated amounts of caspase-3 (Units) and analyzed by Western blotting using anti-AMPK-α1 (2). (B) Caspase-3 has no effect on AMPK activity. Samples from the same experiment as Fig. S4A were assayed for AMPK activity. Note that the results for rat liver AMPK are plotted on the left Y axis and those for bacterially expressed human AMPK on the right Y axis. Results are mean ± SD (n = 2). (C) Purified rat liver AMPK was first depleted of α2-containing complexes by immunoprecipitation, and the remaining α1 complexes were incubated for 2 h with the amounts of bacterially expressed caspase-3 as shown, and analyzed by Wetern blotting using (top) anti-AMPK-α1 (3), (middle) anti-AMPK-pan-β, and (bottom) anti-AMPK-γ1 (2).

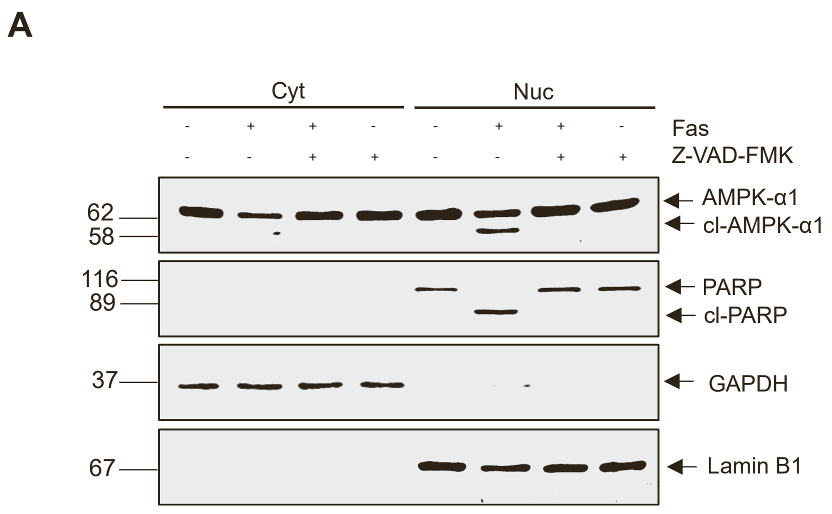

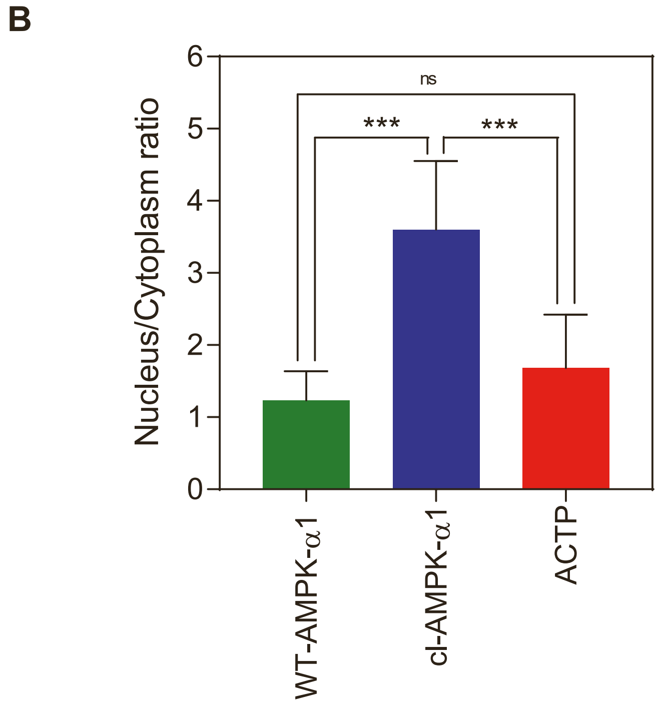

Figure S5. Caspase-cleaved AMPK-α1 prodominantly localizes inside the nucleus, related to Figure 4. (A) Jurkat cells pre-treated with Z-VAD-FMK were treated with anti-Fas. Nuclear and cytoplasmic extractions were prepared and Western blot analysis carried out. (B) As Fig 4H, except that Nuclear/cytoplasmic ratios were quantified; data shown are mean ± SD (n = 3) (***p<0.001 and ns: non-significant)

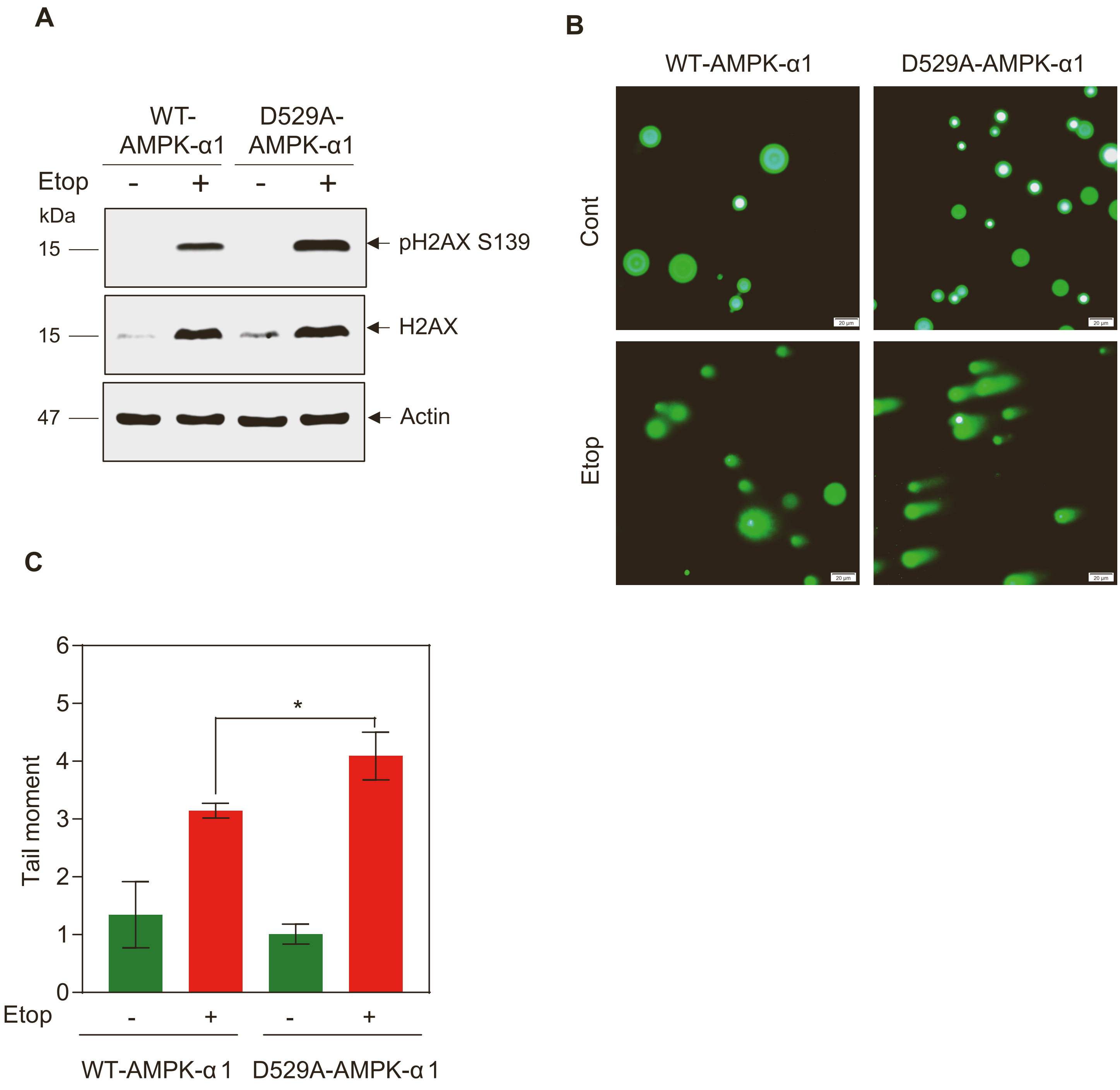

Figure S6. Caspase-cleaved AMPK-α1 protects cells from DNA damage, related to Figure 4. WT and D529A mutant AMPK-α1 transfected cells were treated with Etop. (A) Western blot analysis, (B) comet assay, and (C) tail moment quantification for DNA damage were carried out; data shown are mean ± SD (n = 3) (*p<0.05). (Scale bar, 20 μm).

Treatment	Peptide	Residues	Modified?	Missed cleavages	Queries matched	Ion	Observed m/z	Peptide Mr (exp)	Peptide Mr (calc)
Control	SDSDAEAQGKSSEVSLTSSVTSLDSSPVDLTPR	506-538	no	1	2	$(M+3H)^{3+}$	1118.2017	3351.5832	3351.5802
Control	SDSDAEAQGKSSEVSLTSSVTSLDSSPVDLTPR	506-538	no	1	1	$(M+4H)^{4+}$	839.1569	3352.5986	3351.5802
Control	SDSDAEAQGKSSEVSLTSSVTSLDSSPVDLTPR	506-538	acetyl	1	1	$(M+3H)^{3+}$	1132.2107	3393.6103	3393.5907
Control	SDSDAEAQGKSSEVSLTSSVTSLDSSPVDLTPR	506-538	acetyl	1	1	$(M+4H)^{4+}$	849.6636	3394.6254	3393.5907
Control	SSEVSLTSSVTSLDSSPVDLTPR	516-538	no	0	10	$(M+2H)^{2+}$	1182.5973	2363.1800	2363.1704
Control	SSEVSLTSSVTSLDSSPVDLTPR	516-538	no	0	10	$(M+3H)^{3+}$	788.7326	2363.1761	2363.1704
Control	SSEVSLTSSVTSLDSSPVDLTPR	516-538	no	0	1	$(M+4H)^{4+}$	592.0513	2364.1762	2363.1704
Control	SSEVSLTSSVTSLDSSPVDLTPR	516-538	acetyl	0	6	$(M+2H)^{2+}$	1203.6002	2405.1859	2405.1810
Control	SSEVSLTSSVTSLDSSPVDLTPR	516-538	acetyl	0	7	$(M+4H)^{4+}$	802.7366	2405.1881	2405.1810
Control	PGSHTIEFFEMCANLIK	539-555	oxidation	0	2	$(M+2H)^{2+}$	1025.4798	2028.9450	2028.9387
Control	PGSHTIEFFEMCANLIK	539-555	oxidation	0	5	$(M+3H)^{3+}$	670.6552	2028.9438	2028.9387
Control	PGSHTIEFFEMCANLIK	539-555	dioxidation	0	2	$(M+2H)^{2+}$	1013.9679	2025.9212	2024.9336
Control	PGSHTIEFFEMCANLIK	539-555	dioxidation	0	3	$(M+3H)^{3+}$	675.9872	2024.9399	2024.9336
Control	PGSHTIEFFEMCANLIK	539-555	acetyl plus oxidation	0	1	$(M+3H)^{3+}$	684.6620	2050.9583	2050.9492
Caspase-3	SSEVSLTSSVTSLDSSPVDLTPR	516-538	none	0	1	$(M+2H)^{2+}$	1183.5981	2365.1817	2363.1704

Table S1: **Summary of Mascot Search Results for tryptic peptides from human AMPK complex, related to Figure 3**. Human AMPK complex (bacterially expressed α1β2γ1 complex) incubated either without (Control) or with Caspase-3 (as in Fig. S1A, lanes 1 and 4). Results are limited to C-terminal peptides either containing D529 or C-terminal to that. Where multiple queries matched a single peptide, representative data for observed m/z and Mr (exp) (estimated mass of peptide) are provided.

Note that although the SSEVSLTSSVTSLDSSPVDLTPR peptide was observed in the spectra from the caspase-3 treated sample, it was matched in just one spectrum and with an intensity >180-fold lower than those in the control sample. This may have been due to a very minor degree of cross-contamination between runs on the liquid chromatograph, or between the bands on SDS-PAGE.
